# Synthesis, Evaluation of Enzyme Inhibition and Redox Properties of Potential Dual COX-2 and 5-LOX Inhibitors

**DOI:** 10.3390/ph16040549

**Published:** 2023-04-06

**Authors:** Jelena Bošković, Vladimir Dobričić, Marija Mihajlović, Jelena Kotur-Stevuljević, Olivera Čudina

**Affiliations:** 1 Department of Pharmaceutical Chemistry, University of Belgrade—Faculty of Pharmacy, 11221 Belgrade, Serbia; 2 Department of Medical Biochemistry, University of Belgrade—Faculty of Pharmacy, 11221 Belgrade, Serbia

**Keywords:** dual COX-2 and 5-LOX inhibitors, redox activity, N-hydroxyurea derivatives, 3,5-di-tert-butylphenol derivatives, urea derivatives, “type B hydroxamic acids”

## Abstract

Various dual inhibitors of COX-2 and 5-LOX enzymes have been developed so far in order to obtain more effective and safer anti-inflammatory drugs. The aim of this study was to design and synthesize new dual COX-2 and 5-LOX inhibitors, and to evaluate their enzyme inhibition potential and redox properties. Thirteen compounds (**1**–**13**) were designed taking into account structural requirements for dual COX-2 and 5-LOX inhibition and antioxidant activity, synthesized, and structurally characterized. These compounds can be classified as *N*-hydroxyurea derivatives (**1**, **2** and **3**), 3,5-di-tert-butylphenol derivatives (**4**, **5**, **6**, **7** and **13**), urea derivatives (**8**, **9** and **10**) and “type B hydroxamic acids” (**11** and **12**). COX-1, COX-2 and 5-LOX inhibitory activities were evaluated using fluorometric inhibitor screening kits. The evaluation of the redox activity of newly synthesized compounds was performed in vitro in the human serum pool using redox status tests. The prooxidative score, the antioxidative score and the oxy-score were calculated. Seven out of thirteen synthesized compounds (**1**, **2**, **3**, **5**, **6**, **11** and **12**) proved to be dual COX-2 and 5-LOX inhibitors. These compounds expressed good COX-2/COX-1 selectivity. Moreover, dual inhibitors **1**, **3**, **5**, **11** and **12** showed good antioxidant properties.

## 1. Introduction

Inflammation is a protective process of the immune system against noxious stimuli, and can be classified into an acute form that starts rapidly with symptoms lasting from minutes to a few days, and a chronic form lasting for prolonged periods of several weeks to years. The pathogenesis and progression of various diseases such as cancer, rheumatoid arthritis, autoimmune, cardiovascular and neurodegenerative diseases are closely related to chronic inflammatory processes [1].

Arachidonic acid, AA (5, 8, 11, 14-eicosatetraenoic acid) is the primary precursor in the synthesis of eicosanoids. It is metabolized to prostaglandins and leukotrienes by the enzymes cyclooxygenase (COX) and lipoxygenase (LOX). There are two isoforms of cyclooxygenase: cytoprotective constitutive COX-1 and inducibile COX-2 isoform. Non-steroidal anti-inflammatory drugs (NSAIDs) exert analgesic, antipyretic and anti-inflammatory effects through the inhibition of COX enzymes. They are divided into two groups based on selectivity: traditional non-selective NSAIDs and selective COX-2 inhibitors (coxibs). Non-selective NSAIDs have a wide range of side effects, such as gastrointestinal (GI) disorders, bleeding and renal failure. Selective COX-2 inhibitors have been developed to avoid GI side effects. However, these drugs lead to an increased cardiovascular risk due to decreased endothelial production of the vasodilatory prostacyclin PGI2 and increased levels of vasoconstrictor thromboxane TXA2. Some of them have been withdrawn from the market. The side effects of coxibs occur as a consequence of the potentiation of one biosynthetic pathway by the inhibition of another [2].

Leukotrienes (LTs) as proinflammatory lipid mediators are involved in the development of numerous inflammatory diseases in the body and play the biggest role in cardiovascular and respiratory diseases. After the release of AA from the phospholipid membrane by the enzyme cytosolic phospholipase A2 (cPLA2), AA is further metabolized by the 5-LOX enzyme to LTs. 5-LOX, as a crucial enzyme in LTs production, represents an important target for improved therapy. There are four classes of 5-LOX enzyme inhibitors: (1) redox active inhibitors, (2) iron chelators (chelate the active site iron), (3) non-redox, competitive, reversible enzyme inhibitors and (4) inhibitors that could act in an allosteric mode [3].

The COX-2 enzyme is often expressed in many types of cancer, and it plays a role in the formation or promotion of carcinogenesis, as well as in the occurrence of resistance to radiotherapy and chemotherapy. This enzyme promotes proliferation, angiogenesis, invasion and metastasis, and inhibits apoptosis, in cancer cells. It is assumed that using COX-2 inhibitors as adjuvant drugs would be a promising strategy in cancer treatment. The 5-LOX enzyme produces leukotriene B4 (LTB4) and 5-hydroxyeicosatetranoic acid (5-HETE), which express cancer growth-promoting effects. Molecular pathobiological studies have concluded that the inflammatory process plays an important role in the development of colorectal cancer, in which elevated levels of the enzymes COX-2 and 5-LOX occur. Based on the conclusions of the study, COX-2 and 5-LOX enzymes are important targets in the development of drugs that would be used in the treatment of colorectal cancer, which has been linked to more than 600,000 deaths a year, and this incidence rate is rising in most developing countries. The poor antitumor efficacy of COX-2 inhibitors against certain tumor types can be explained by the fact that the blockade of one enzyme pathway potentiates another, from which it can be concluded that the blockade of both pathways (inhibition of COX-2 and 5-LOX enzymes) is a good approach to effective colorectal cancer therapy [4,5,6,7,8,9].

There is a balance between the production and neutralization of free radicals in biological systems. Oxidative stress represents a disturbance in the balance between oxidation and reduction processes that results in the excessive production of free radicals that the homeostatic mechanisms of cells are unable to neutralize. Reactive oxygen compounds (ROS, i.e., reactive oxygen species) and reactive nitrogen compounds (RNS, i.e., reactive nitrogen species) have numerous physiological functions in cells, such as defending the organism against infectious agents, the regulation of cell proliferation and death, the regulation of metabolism, and intercellular communication. When overproduced, ROS and RNS damage DNA, proteins and lipids and lead to various diseases [10]. Cell antioxidants are classified as enzymatic (superoxide dismutase (SOD), glutathione peroxidase and reductase (GPx), catalase (CAT)) and non-enzymatic (biomolecules, vitamins and exogenous nutrients). They play a role in antioxidant protection by eliminating free radical species, while they become oxidized themselves. A good antioxidant molecule has the ability to transform into a new free radical species, which has low activity and lower damaging potential than reactive molecules when neutralization is performed [11]. Oxidative stress plays an important role in the pathogenesis of various diseases, such as cancer, cerebrovascular diseases and Alzheimer’s disease [12,13]. On the other hand, oxidative stress is part of the regular metabolism, and involves upkeeping of low levels of oxidants, thus placing oxidative stress on basic life processes [14]. A connection between inflammation and oxidative stress is considered to exist, because protein oxidations caused by ROS may influence the release of inflammatory mediators [15]. It is also well known that mutual potentiation between inflammation and oxidative stress is realized through different transcription factors, and primarily through the redox-sensitive nuclear factor kappa B (NF-κB). This activation increases proinflammatory genes’ expression and further increases inflammatory proteins synthesis [16].

Various dual COX-2 and 5-LOX inhibitors with diverse structural characteristics have been developed and tested. The design of potential dual inhibitors is based on the modification of NSAIDs by introducing LOX pharmacophores; this has been explored through SAR and QSAR studies, as well as molecular docking studies (computational techniques were used in order to develop new scaffolds). Di-tert-butylphenol derivatives, pyrrolizine-based derivatives, modified NSAIDs and modified coxibs are the most extensively investigated groups of dual COX-2 and 5-LOX inhibitors. Tepoxalin (hydroxamic acid) is approved for veterinary use, while licofelone (pyrrolizine-based dual inhibitor) has undergone a phase III clinical trial for human use [2,17].

The aim of this study was the synthesis, physicochemical characterization and biological testing (in vitro evaluation of enzyme inhibition potential and redox properties) of novel compounds with potential dual COX-2 and 5-LOX inhibitory activity.

## 2. Results and Discussion

### 2.1. Design and Synthesis

**Scheme 1:**
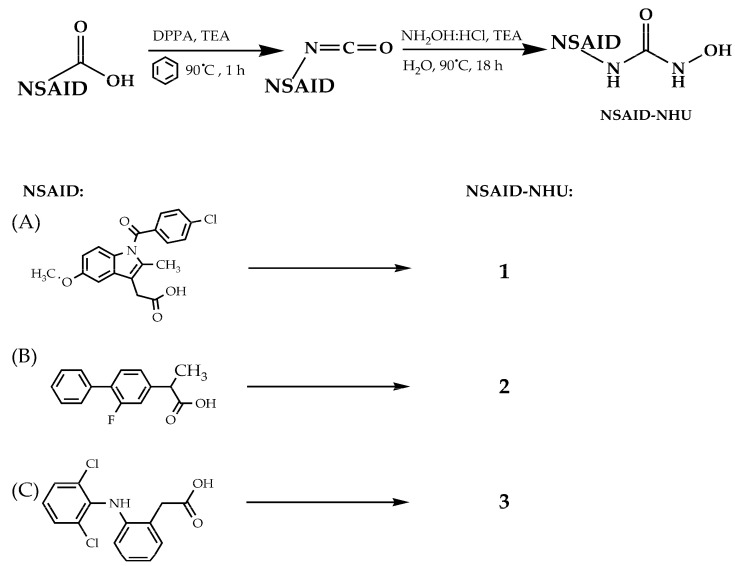
Synthesis of *N*-hydroxyurea derivatives: (**A**) compound **1**, (**B**) compound **2** and (**C**) compound **3**.

It was reported that the replacement of the carboxylate group of NSAIDs with the *N*-hydroxyurea (NHU) group would provide 5-LOX inhibitory activity, while COX-2 inhibitory activity could be either lost or retained [18]. Three NSAIDs ((a) indomethacin, (b) flurbiprofen and (c) diclofenac, Scheme 1) were selected as starting compounds in the synthesis of N-hydroxyurea derivatives (**1**, **2** and **3**, Scheme 1). These NSAIDs have different COX inhibitory potentials, and they were selected in order to investigate the influence of the introduction of the 5-LOX pharmacophore on the pre-existing COX inhibitory potential [19]. NSAID isocyanate, formed as a product of the Curtius rearrangement reaction of the corresponding NSAID with diphenyl phosphorazidate (DPPA), in further reaction with hydroxylamine gives the product NSAID-NHU.

**Scheme 2:**
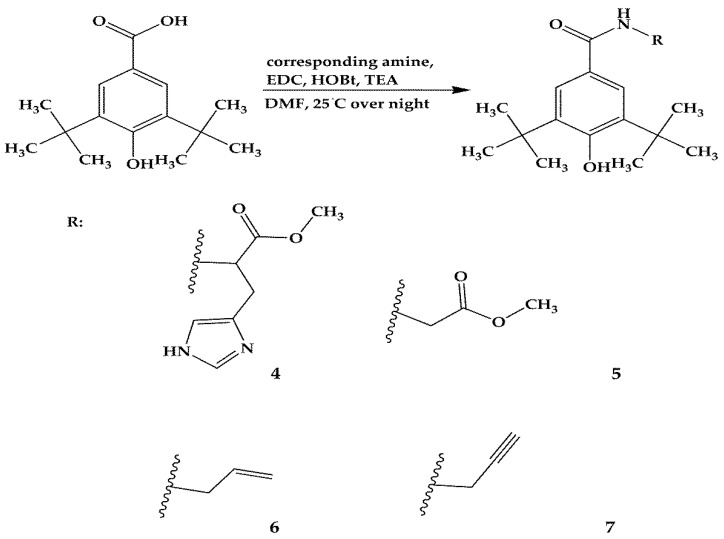
Synthesis of 3,5-di-tert-butyl-4-hydroxybenzoic acid derivatives (amides).

Previously performed SAR studies showed that 3,5-di-tert-butyl-4-hydroxy-benzene substituted in position C1 acts as an optimum scaffold for dual COX-2 and 5-LOX activity. The phenol group expresses antioxidant properties, and its presence is important for anti-inflammatory activity [17,20]. Considering that the derivation of the carboxyl group of 3,5-di-tert-butyl-4-hydroxybenzoic acid has been extensively studied and certain derivatives have been highlighted as good anti-inflammatory agents [17], our aim was to investigate the effect of introducing amino acids into the side chain (derivatives **4** and **5**) on COX-2’s and 5-LOX’s inhibitory activity. Compound **5** (*N*-(3,5-di-tert-butyl-4-hydroxybenzoyl)glycine methyl ester) was previously reported as a starting material for the synthesis of methyl 5-(3,5-di-tert-butyl-4-hydroxyphenyl)-1-tetrazoleacetate as an aldose reductase inhibitor [21]. Derivatives **6** and **7** were designed and synthesized to investigate the effect of introducing unsaturated (double and triple) bonds into the side chain on COX-2 and 5-LOX’s inhibitory activity, considering that a derivative with a similar structure (tebufelone) led to excellent dual COX-2 and 5-LOX inhibitory activity, and is currently in the clinical trials stage [17]. Derivatives **4**, **5**, **6** and **7** (Scheme 2) were synthesized in reactions of 3,5-di-tert-butyl-4-hydroxybenzoic acid with L-histidine methyl ester, glycine methyl ester, allylamine and propargylamine, respectively. Syntheses were performed in the presence of 1-ethyl-3-(3-dimetylaminopropyl)carbodiimide (EDC), hydroxybenzotriazole (HOBt) and triethylamine (TEA) according to procedures in the literature [22].

**Scheme 3:**
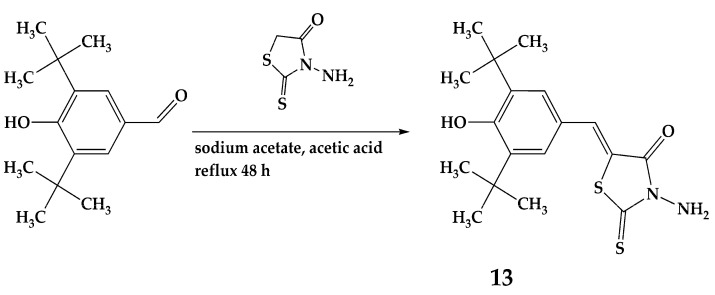
Synthesis of compound **13**.

Derivatives of 2,6-di-tert-butylphenol linked to various heterocyclic rings (directly or by carbon chain) have been widely studied as potential dual COX-2 and 5-LOX inhibitors [23]. However, bicyclic 2,6-di-tert-butylphenol derivatives have shown low bioavailability and aqueous solubility as a result of high lipophilicity. It was shown that the addition of ionizing groups to heterocyclic ring leads to better bioavailability. Benzylideneoxazoles-tiazole and -imidazole derivatives were synthesized and many of them were dual inhibitors, while some of them showed activity in animal inflammation models after oral administration. Compound **13** belongs to the group of 2,6-di-tert-butylphenol derivatives with the thiazolidine ring in the side chain [24]. This compound was synthesized by the Knoevenagel condensation of 3,5-di-tert-butyl-4-hydroxybenzaldehyde with 3-aminorhodanine (Scheme 3).

**Scheme 4:**
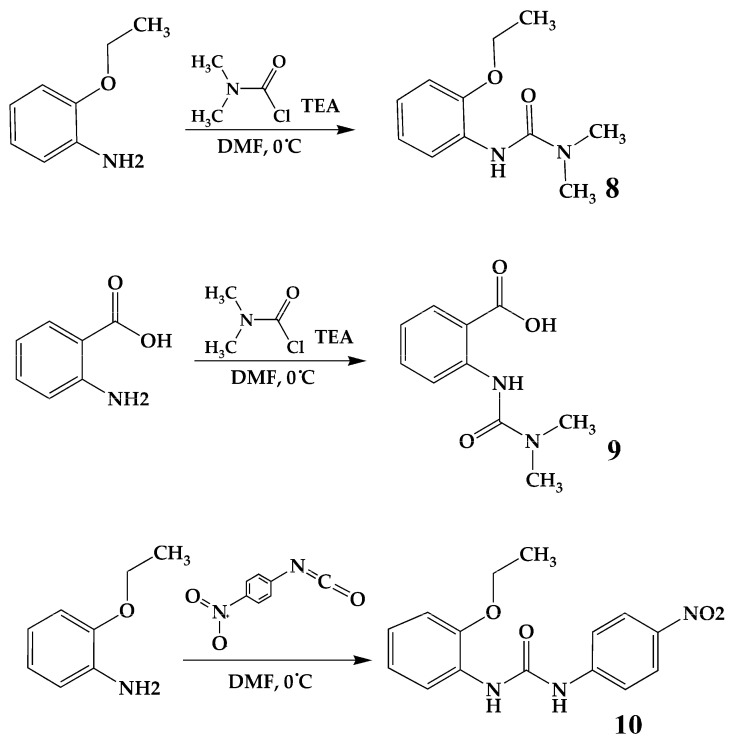
Synthesis of compounds **8**, **9** and **10**.

The introduction of a urea group could be a promising strategy to obtain 5-LOX inhibitors [25]. This group can also be found in compounds that possess dual inhibitory activity towards COX-2 and soluble epoxide hydrolase (sEH) [26]. Urea derivatives **8**, **9** and **10** (Scheme 4) were synthesized according to the procedure in the literature [27]. Compounds **8** and **9** were synthesized in the reaction of dimethylcarbamoyl chloride with 2-ethoxyaniline and anthranilic acid, respectively [27]. Compound **8** was previously synthesized and tested for herbicidal activity [28]. Compound **9** was previously synthesized in a similar manner in the reaction of anthranilic acid with N, *N*-dimethylcarbamoyl chloride in the presence of triethylamine, to examine the influence of the base on the formation of the final product [29]. Compound **9** was also previously synthesized in the reaction of isatoic anhydride and dimethylamine [30]. Compound **10** was synthesized in the reaction of 2-ethoxyaniline with 4-nitrophenyl isocyanate.

**Scheme 5:**
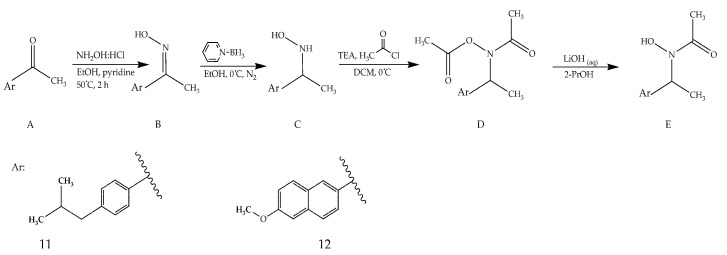
Synthesis of compounds **11** and **12**.

Potential 5-LOX inhibitors were developed by incorporating hydroxamic acid into the structures of various molecules. Studies showed that compounds possessing small groups attached to carbonyl with hydroxamate moiety and substantial groups attached to nitrogen (“type B hydroxamic acids”) exhibit better pharmacokinetic profiles than classic hydroxamic acids. Compounds **11** and **12** were synthesized according to the procedure in the literature (Scheme 5). NSAID (ibuprofen, naproxen)-like ketones were selected as starting compounds that were further transformed into oxime (in this way, the final product retains NSAID’s scaffold, which gives it the potential to inhibit COX-2 enzyme). The obtained oxime was reduced with a borane–pyridine complex, and the resulting hydroxylamine was treated with acetyl chloride to give N,O-diacetate as a product. The final product was obtained after the selective elimination of O-acetate with lithium-hydroxide (LiOH). These compounds were primarily synthesized as 5-LOX inhibitors [31].

NMR spectra of all synthesized compounds are presented in Figures S1–S13 (Supplementary material).

### 2.2. In Vitro COX-1, COX-2 and 5-LOX Inhibition Assay

The ability of newly synthesized compounds **1–13** to inhibit COX-1, COX-2 and 5-LOX enzymes was tested using fluorimetric screening kits. The results are presented as IC_50_ ± standard error (µM) and compared to the commercially available COX-2 inhibitor celecoxib and 5-LOX inhibitor zileuton (Table 1).

All of the tested *N*-hydroxyurea derivatives (compounds **1**, **2** and **3**) have dual COX-2 and 5-LOX inhibitory activities. It can be seen that analogs of the potent NSAIDs flurbiprofen (compound **2**) and diclofenac (compound **3**) showed stronger dual COX-2 and 5-LOX inhibitory activities than the analog of indomethacin (compound **1**). In addition, compounds **2** and **3** were the most potent dual COX-2 and 5-LOX inhibitors among all synthesized compounds.

In the class of 3,5-di-*tert*-butyl phenol derivatives (compounds **4**–**7** and **13**), compounds **5** and **6** exhibited relatively good dual COX-2 and 5-LOX inhibitory activity. Compound **6** had the best COX-2 inhibitory activity, while compounds **4** and **5** showed the best 5-LOX inhibitory activity. These results prove that the substituent in position C1 of 3,5-di-tert-butyl-4-hydroxy-benzen derivatives has an important influence on COX-2 and 5-LOX inhibitory activity.

Both of the “type B hydroxamic acid” derivatives proved to be dual COX-2 and 5-LOX inhibitors with similar 5-LOX inhibitory activities (**11**: IC_50_ = 1.04 ± 0.22 µM; **12**: IC_50_ = 1.29 ± 0.10 µM), while **11** had a significantly better COX-2 inhibitory activity than **12** (36.18 ± 3.08 µM vs. 83.42 ± 4.37 µM).

Neither of the urea derivatives (**8**, **9** and **10**) can be considered dual inhibitors, but compounds **8** and **10** were shown to possess significant 5-LOX inhibitory activity (with IC_50_ values 14.52 ± 3.62 and 13.01 ± 2.67, respectively).

All compounds with dual COX-2 and 5-LOX inhibitory activity (**1**, **2**, **3**, **5**, **6**, **11** and **12**) were also tested for COX-1 inhibitory activity. None of them reached 50% enzyme inhibition at 100 µM concentration, so it could be concluded that they are weak COX-1 inhibitors and have satisfactory selectivity towards COX-2 enzyme.

### 2.3. Evaluation of Redox Activity of Synthesized Compounds

**Figure 1:**
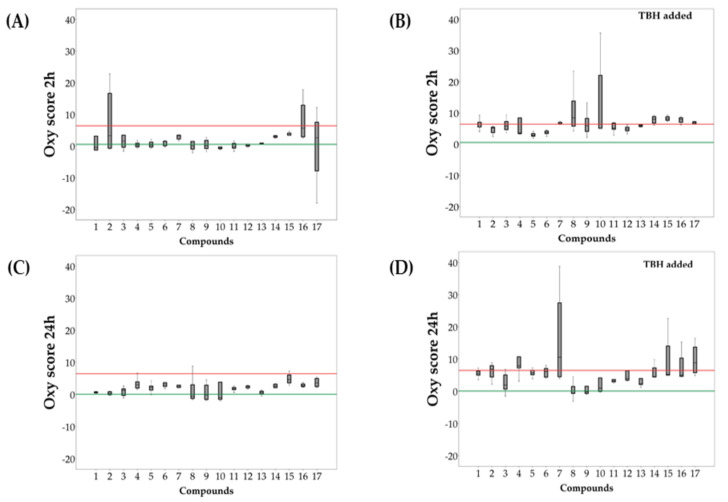
Obtained oxy scores (Y axes) of tested compounds: (**A**) after 2 h incubation, (**B**) after 2 h incubation, with added TBH, (**C**) after 24 h incubation, (**D**) after 24 h incubation with added TBH. Compounds (X axes): **1**–**13** newly synthesized compounds, 14 BHT, 15 urea, 16 celecoxib, 17 zileuton; the OS of antioxidant Trolox is presented as a green line; the OS of TBH is presented as a red line.

The evaluation of the redox activity of newly synthesized compounds **1**–**13** was performed in vitro in the human serum pool using redox status tests (with and without egzogenous oxidant-tert-butyl hydroperoxide (TBH)), such as TOS (total oxidative status), PAB (prooxidative–antioxidative balance), TAS (total antioxidative status) and SHG (total protein’s sulfhydril groups content). Prooxidative score, antioxidative score and oxy score (OS) were further calculated. Five different dilutions of each compound were tested. The obtained results indicate that there was no dose dependence reactivity, so the results are presented as average values of five concentrations. The obtained results (OS values obtained after 2 h and 24 h incubation and before and after TBH addition) are presented in Figure 1. Statistically significant differences before and after TBH addition (*p* values) and statistically significant differences between 2 h and 24 h incubation for the same samples, as well as the OS values obtained for the Trolox (standard antioxidant) and TBH (prooxidant), are presented in Table 2.

Butylated hydroxytoluene (BHT), urea, celecoxib and zileuton were tested as standard substances in order to make comparisons with the tested compounds.

One of the most important classes of antioxidants is the phenolic antioxidants. BHA and BHT are phenolic antioxidants that contain a phenolic ring with di-tert-butyl groups responsible for huge antioxidant potential. BHA is a mixture of two isomers: 3-tert-butyl-4-methoxy phenol and 2-tert-butyl-4-methoxy phenol. The two-isomer structure is considered to yield a better antioxidant. BHT is a di-tert-butyl phenol that is extensively used as an antioxidant in the pharmaceutical, chemical and food industries. The influence of the substituents on antioxidant potency has been investigated in many studies. It is considered that electron-donating p-substituents stabilize the phenoxyl radical and improve its antioxidant properties [32]. Five compounds that contain di-tert-butyl phenol pharmacophore (**4**, **5**, **6**, **7** and **13**) have been tested. Four of them (**4**, **5**, **6** and **13**) showed lower OS than BHT after 2 h incubation, indicating that those compounds are better antioxidants. After 24 h of incubation, **5**, **7** and **13** showed lower OS than BHT. There is a statistically significant difference between the results obtained after 2 h incubation and those obtained after 24 h incubation in the case of **4** and **5** (without added TBH), which implies that their antioxidant activities slightly decreased over time. There is also a statistically significant difference between results obtained after 2 h incubation and those obtained after 24 h incubation in the case of **13** (with added TBH) (5.89 (5.63–6.14) vs. 2.35 (2.10–3.92), *p* < 0.037), which indicates that as the incubation time increased, the compound more effectively resisted the effects of prooxidant. There is a significant difference between samples with and without TBH added in almost all cases (OS values significantly increase after TBH addition), even in the case of BHT, so it could be concluded that the synthesized compounds show similar behaviors to BHT in the presence of TBH (in both cases, after 2 h and 24 h incubation). It is considered that compounds better resist the effects of exogenous prooxidant in cases where there are no significant differences between OS values from before and after TBH addition. All compounds are ranked based on their OS median values, which can be categorized into distinct groups (without TBH, with added TBH, 2 h incubation, 24 h incubation), and compounds **5** and **13** are the most promising antioxidants from this class.

Previous studies have shown that urea also has antioxidant activity. It shows some superiority in comparison to other natural or synthetic antioxidants because of its ability to easily pass through membranes and it has a low molecular weight. Urea is a biomolecule, a non-protein nitrogen-containing compound that is a metabolic end-product of nitrous products’ catabolism, with diagnostic significance for renal disease. Its structurally reduced characteristics yield certain cardioprotective effects, due to its ability to counteract oxidative stress. The urea derivatives hydroxyurea and dymethylurea showed antioxidant effects too [33]. The antioxidant properties of urea have been tested in this study, and the obtained results (OS) indicate that there was no significant difference between the 2 h- and 24 h-incubated samples. There was statistically significant difference between urea samples with and without TBH after 2 h incubation (OS value increased), while after 24 h incubation, there was no statistically significant difference (*p* = 0.149). It is assumed that with a longer incubation time, urea more effectively opposes the effect of the prooxidant (TBH). The urea derivatives (**8**, **9** and **10)** showed similar behaviors to urea. These compounds had lower OS than urea, and they could be assumed to act as potent antioxidants. In the case of compound **8**, it could be observed that the incubation time had a significant effect on its ability to resist the prooxidant effect. The *N*-hydroxy urea derivatives (compounds **1**, **2** and **3**) had antioxidant effects similar to that of urea. Compound **1** had a negative OS value after 2 h incubation, and there was no significant difference after 24 h incubation. Compound **3** yielded lower OS values than urea, and after 24 h incubation the difference between samples with and without TBH (better resistance to the effects of prooxidant) was lost (*p* = 0.226).

Compounds **11** and **12** had lower OS values than urea. The OS values increased with the increasing incubation time, and there were statistically significant differences between the results obtained after 2 h incubation and those obtained after 24 h incubation. The antioxidant activity slightly decreased as the time passed. The ability of compound **11** to counteract prooxidants increased as the incubation time passed.

In the cases of compounds **3**, **8**, **9** and **10** (24 h incubation), there was no statistically significant increase in the OS value after the addition of TBH, which indicates that some kind of equilibrium was reached as the time passed, whereby the compounds could better resist the effects of prooxidants.

The selective COX-2 inhibitor (celecoxib) and 5-LOX inhibitor (zileuton) showed weaker antioxidant activities than the synthesized compounds. The synthesized compounds could be ranked according to their antioxidant potential (OS median values) in the following order: **10** > **9** > **8** > **11** > **1/5** > **12/13** > **3** > **4** > **2** > **6** > **7**.

Wilcoxon’s paired test was used to determine whether there were statistically significant differences between all the tested compounds. Statistically significant differences are presented in Tables S1–S4 (Supplementary material).

## 3. Materials and Methods

### 3.1. General

All chemicals, reagents and solvents used for synthesis were purchased from Sigma-Aldrich (St. Louis, MO, USA), Acros Organics (Geel, Belgium), Thermo Fisher Scientific (Waltham, MA, USA) and VWR Chemicals BDH (VWR International—Radnor, PA, USA). The purity of the synthesized compounds ranged from 95.7 to 99.9% and was determined using an Aglient 1200 HPLC chromatograph (Agilent Technologies, Santa Clara, CA, USA), equipped with a binary pump, a manual injector (20 µL) and a PDA detector. Melting points were determined on the Boetius PHMK 05 apparatus (VEB Wagetechnik Rapido, Radebeul, Germany). The IR spectra were recorded on a Thermo Nicolet 6700 FT-IR spectrophotometer using the ATR technique (Thermo Fisher Scientific, Waltham, MA, USA). The NMR spectra were recorded on a Bruker Ascend 400 (400 MHz) spectrometer (Bruker, Billerica, MA, USA). Chemical shifts are given in parts per million downfield from tetramethylsilane as the internal standard. The mass spectra were recorded on a TSQ Quantum Access MAX triple quadrupole mass spectrometer equipped with electrospray ionization source (ESI) (Thermo Fisher Scientific, Waltham, MA, USA). Exact mass measurements were performed using a Thermo Scientific LTQ Orbitrap XL high-resolution mass spectrometer (Thermo Fisher Scientific, Waltham, MA, USA).

### 3.2. Synthesis of Compounds 1–13

#### 3.2.1. Synthesis of Compounds **1**–**3**

Indomethacin, flurbiprofen and diclofenac (0.84 mmol, 1 eq) were dissolved in benzene (5 mL). Triethylamine (0.84 mmol, 1 eq) and DPPA (0.84 mmol, 1 eq) were added to the solution. The mixture was heated at 90 °C for 1 h. A solution of hydroxylamine HCl (1.68 mmol, 2 eq) and triethylamine (1.68 mmol, 2 eq) in water (0.3 mL) was added and the reaction mixture was stirred at 90 °C for 18 h. The mixture was cooled to room temperature. The product was further purified by preparative TLC using dichloromethane-methanol 9.5:0.5 *v*/*v* (**1**, **2**) or 9:1 *v*/*v* (**3**).

*N’*-hydroxy-*N*-[(1-(4-chlorobenzoyl)-5-methoxy-2-methyl-3-indolyl)methyl]urea (**1**). Light-yellow crystalline solid. Yield: 21%. Melting point: 97–99 °C. ^1^H NMR (400 MHz, CDCl_3_) δ ppm 2.42 (3H, s, CH_3_), 3.82 (3H, s, OCH_3_), 4.55 (2H, d, J = 5.6, CH_2_), 5.99 (1H, s, R-NH-C(=O)NHOH), 6.14 (1H, s, R-NH-C(=O)NHOH), 6.66–7.67 (7H, m, Ar-H), 6.80–6.84 (1H, m, OH). ^13^C NMR (100.6 MHz, CDCl_3_) δ ppm 13.08, 33.67, 55.84, 101.50, 111.87, 114.98, 115.84, 129.21, 130.00, 131.00, 131.24, 133.69, 136.34, 139.56, 156.12, 161.07, 168.43. *m*/*z* = 388.3 (M^+^ +1), 312.17, 139.07, 111.12, 129.16, 355.17, 93.22. IR (ATR) υ_max_ (cm^−1^): 752.46, 1114.01, 1220.50, 1361.33, 1477.36, 1548.26, 1654.49, 2937.15, 3207.02, 3387.09. HRMS-HESI: [M+H]^+^ calculated = 388.10586; observed = 388.10549.

*N*’-hydroxy-*N*-(2-ethyl-(3-fluoro-4-phenylphenyl))urea (**2**). White crystalline solid. Yield: 36.5%. Melting point: 121.5–123 °C. ^1^H NMR (400 MHz, CDCl_3_) δ ppm 1.43 (3H, d, J = 6.8, CH_3_), 4.89 (1H, t, J = 7.2, CH), 7.12 (1H, d, J = 8.4, R-NH-C(=O)-NHOH), 7.25–7.54 (8H, m, Ar-H), 8.40 (1H, s, R-NH-C(=O)-NHOH), 8.65 (1H, s, OH). ^13^C NMR (100.6 MHz, CDCl_3_) δ ppm 21.69, 47.06, 112.85–113.09, 121.97–122.00, 127.06, 127.96–128.12, 129.77–129.81, 134.50, 125.58–125.71, 147.24–147.31, 157.13, 159.57, 159.97. *m*/*z* = 275.3 (M^+^ +1), 199.19, 178.19, 183.18, 152.19, 77.28, 176.14. IR (ATR) υ_max_ (cm^−1^): 690.67, 764.75, 826.45, 1076.97, 1411.28, 1548.56, 2161.77, 3250.36, 3407.48. HRMS-HESI: [M+H]^+^ calculated = 275.11903; observed = 275.11883.

*N*’-hydroxy-*N*-[(2-(2,6-dichloroanilino)phenyl)methyl]urea (**3**). White crystalline solid. Yield: 24%. Melting point: 163–165 °C. ^1^H NMR (400 MHz, DMSO) δ ppm 4.34 (2H, d, J = 6.4, CH_2_), 6.11 (1H, d, J = 8, H-f), 6.77 (1H, t, J = 7.2, H-d), 7.01 (1H, t, J = 7.6, H-f’), 7.25 (2H, m, H-c, H-e), 7.42 (1H, t, J = 6, R-NH-C(=O)-NHOH), 7.54 (2H, d, J = 8, H-a’, H-e’), 7.90 (1H, s, NH), 8.57 (1H, s, R-NH-C(=O)-NHOH), 8.68 (1H, s, OH). ^13^C NMR (100.6 MHz, CDCl_3_) δ ppm 40.00, 114.27, 119.60, 126.94, 128.15, 129.57, 130.89, 127.29, 132.64, 137.55, 143.19, 162.49. *m*/*z* = 326.2 (M^+^ +1), 214.06, 250.04, 179.09, 238.07, 178.07, 151.22. IR (ATR) υ_max_ (cm^−1^): 748.44, 1150.93, 1268.44, 1446.45, 1552.85, 1622.11, 2162.30, 3293.12, 3389.55. HRMS-HESI: [M+H]^+^ calculated = 326.04576; observed = 326.04542.

#### 3.2.2. Synthesis of Compounds **4**–**7**

3,5-di-tert-butyl-4-hydroxybenzoic acid (0.42 mmol, 1 eq) was dissolved with the corresponding amino acid (L-histidine methyl ester dihydrochloride for compound **4**; glycine methyl ester for compound **5**), allylamine (for compound **6**) or propargylamine (for compound **7**) (0.5 mmol, 1.2 eq) in dimethylformamide (DMF, 6 mL) at room temperature. EDC (0.63 mmol, 1.5 eq), HOBt (0.63 mmol, 1.5 eq) and TEA (0.84 mmol, 2 eq) were added. The reaction mixture was stirred overnight at room temperature and the product was further purified by preparative TLC using dichloromethane-methanol 9.5:0.5 *v*/*v* and 9:1 *v*/*v* (**4**), dichloromethane-methanol 20:0.5 *v*/*v* (**5**), dichloromethane-methanol 9.5:0.5 *v*/*v* (**6**) or dichloromethane-methanol 9:1 *v*/*v* (**7**).

2-(3,5-di-tert-butyl-4-hydroxy-benzoylamino)-3-(1H-imidazol-4-yl)-propionic acid methyl ester (**4**). Light-gray crystalline solid. Yield: 28%. Melting point: 117–119 °C. ^1^H NMR (400 MHz, CDCl_3_) δ ppm 1.47 (18H, s, 2 × (C(CH_3_)), 3.20 (2H, m, CH_2_), 3.70 (3H, s, CH_3_), 4.92 (1H, m, R-CH), 5.55 (1H, s, NH(imidazol)), 6.84 (1H, s, H-d’), 7.56 (1H, s, H-b’), 7.76 (2H, s, H-c, H-e), 8.19 (1H, s, C(=O)NH). ^13^C NMR (100.6 MHz, CDCl_3_) δ ppm 29.14, 30.16, 34.45, 52.34, 52.91, 115.23, 124.62–124.79, 134.92, 135.75, 157.03, 167.76, 172.35. *m*/*z* = 402.2 (M^+^ +1), 233.02, 170.02, 217.04, 110.11, 149.07, 370.15. IR (ATR) υ_max_ (cm^−1^): 657.57, 1103.17, 1236.34, 1433.29, 1532.94, 1740.73, 2161.86, 2955.52, 3213.79. HRMS-HESI: [M+H]^+^ calculated = 402.23873; observed = 402.23797.

(3,5-di-tert-butyl-4-hydroxy-benzoylamino)-acetic acid methyl ester (**5**). White crystalline solid. Yield: 71%. Melting point: 130–132 °C. ^1^H NMR (400 MHz, CDCl_3_) δ ppm 1.46 (18H, s, 2 × (C(CH_3_)), 3.80 (3H, s, CH_3_), 4.24 (2H, d, J = 5.2, CH_2_), 5.57 (1H, s, OH), 6.52 (1H, s, NH), 7.64 (2H, s, H-c, H-e). ^13^C NMR (100.6 MHz, CDCl_3_) δ ppm 30.15, 34.42, 41.74, 52.40, 124.32, 124.90, 135.98, 157.12, 168.20, 170.85. *m*/*z* = 320.3 (M^+^ −1), 260.13, 204.11, 288.13, 244.12, 232.12, 233.16. *m*/*z* = 322.5 (M^+^ +1), 232.45, 216.30, 233.50, 321.73, 147.86, 145.95. IR (ATR) υ_max_ (cm^−1^): 770.60, 888.59, 1203.90, 1433.65, 1639.49, 1745.67, 2161.37, 2954.27, 3298.14, 3619.53. HRMS-HESI: [M+H]^+^ calculated = 322.20128; observed = 322.20088.

*N-allyl-3,5-di-tert-butyl-4-hydroxy-benzamide* (**6**). White crystalline solid. Yield: 80%. Melting point: 182–183.5 °C. ^1^H NMR (400 MHz, CDCl_3_) δ ppm 1.46 (18H, s, 2 × (C(CH_3_)), 4.08 (2H, s, CH_2_), 5.16–5.28 (2H, m, R-CH=CH_2_), 5.55 (1H, s, NH), 5.95 (1H, m, R-CH=CH_2_), 6.08 (1H, s, OH), 7.61 (2H, s, Ar-H). ^13^C NMR (100.6 MHz, CDCl_3_) δ ppm 30.18, 34.43, 42.42, 116.34, 124.10, 125.72, 134.66, 135.95, 156.82, 168.05. *m*/*z* = 290.1 (M^+^ +1), 232.98, 176.95, 234.03, 216.95, 160.95, 149.02. IR (ATR) υ_max_ (cm^−1^): 720.05, 923.43, 1143.47, 1233.10, 1433.61, 1539.74, 1630.31, 2952.27, 3243.78, 3624.47. HRMS-HESI: [M+H]^+^ calculated = 290.21146; observed = 290.21111.

*3,5-di-tert-butyl-4-hydroxy-N-prop-2-ynyl-benzamide* (**7**). White crystalline solid. Yield: 86.5%. Melting point: 178.5–180 °C. 1H NMR (400 MHz, CDCl_3_) δ ppm 1.46 (18H, s, 2 ×(C(CH3)), 2.27 (1H, s, CH), 4.24–4.26 (2H, m, NHCH_2_), 5.57 (1H, s, OH), 6.14 (1H, s, NH), 7.60 (2H, s, Ar-H). 13C NMR (100.6 MHz, CDCl_3_) δ 29.69, 30.16, 34.43, 71.58, 79.93, 124.23, 124.95, 135.99, 157.08, 167.80. *m*/*z* = 288.2 (M^+^ +1), 232.19, 177.17, 203.16, 217.15, 161.16, 176.15. IR (ATR) υ_max_ (cm^−1^): 3615.87, 3239.49, 2956.82, 1632.64, 1545.80, 1308.20, 1232.59, 1117.05, 899.31, 683.44. HRMS-HESI: [M+Na]^+^ calculated = 310.17775; observed = 310.17805.

#### 3.2.3. Synthesis of Compounds **8** and **9**

2-ethoxyaniline (o-phenetidin) (for synthesis of **8**) or anthranilic acid (for synthesis of **9**) (7.66 mmol, 1 eq) was dissolved in 15 mL of dichloromethane and the solution was cooled to 0 °C. Subsequently, TEA (7.66 mmol, 1 eq) and dimethylcarbamoyl chloride (9.19 mmol, 1.2 eq) were added. The mixture was kept overnight at room temperature. The reaction mixture was purified by preparative TLC using cyclohexane-ethyl acetate 6:3 *v*/*v* (compound **8**) or using cyclohexane-ethylacetate 1:1 *v*/*v*, then chloroform-methanol 9:1 *v*/*v* (compound **9**).

3-(2-ethoxy-phenyl)-1,1-dimethyl-urea (**8**). Light-brown crystalline solid. Yield: 7%. Melting point: 71.5–72.5 °C. ^1^H NMR (400 MHz, CDCl_3_) δ ppm 1.44 (3H, t, J = 6.8, CH_3_), 3.04 (6H, s, R-N-(CH_3_)_2_), 4.09 (2H, m, CH_2_), 6.84–6.93 (3H, m, Ar-H), 7.17 (1H, s, NH), 8.19 (1H, m, Ar-H). ^13^C NMR (100.6 MHz, CDCl_3_) δ ppm 14.97, 36.26, 64.12, 110.73, 118.67, 121.17, 121.72, 129.27, 146.83, 155.51. *m*/*z* = 209.1 (M^+^ +1), 72.28, 164.12, 46.44, 56.26, 136.16, 44.45. IR (ATR) υ_max_ (cm^−1^): 737.72, 1043.13, 1245.73, 1449.80, 1531.35, 1666.80, 2931.29, 2978.69, 3447.16. HRMS-HESI: [M+Na]^+^ calculated = 231.11040; observed = 231.11028.

2-(3,3-dimethyl-ureido)-benzoic acid (**9**). Light-brown crystalline solid. Yield: 12%. Melting point: 158–159.5 °C. ^1^H NMR (400 MHz, DMSO) δ ppm 2.98 (6H, s, R-N-(CH_3_)_2_)), 6.97–8.48 (4H, m, Ar-H), 10.83 (1H, s, NH), 13.49 (1H, s, R-COOH). ^13^C NMR (100.6 MHz, DMSO) δ ppm 35.20, 113.78, 117.94, 119.75, 130.37, 133.42, 142.78, 154.13, 169.74. *m*/*z* = 209.1(M^+^ +1), 120.11, 72.31, 191.07, 92.16, 146.05, 90.16. IR (ATR) υ_max_ (cm^−1^): 696.62, 747.54, 1030.68, 1233.39, 1448.61, 1532.39, 1601.52, 2606.81, 2922.45. HRMS-HESI: [M+Na]^+^ calculated = 231.07401; observed = 231.07413.

#### 3.2.4. Synthesis of Compound **10**

To the solution of 2-Ethoxyaniline (0.73 mmol, 1 eq) in dimethylformamide (1 mL), 4-Nitrophenyl isocyanate (1.46 mmol, 2 eq) was added at 0 °C. The reaction mixture was stirred overnight at room temperature. The product was purified by preparative TLC using chloroform:methanol 99:1 *v*/*v*.

1-(2-ethoxy-phenyl)-3-(4-nitro-phenyl)-urea (**10**). Yellow crystalline solid. Yield: 23.5%. Melting point: 178–181 °C. ^1^H NMR (400 MHz, DMSO) δ ppm 1.42 (3H, t, J = 8, CH_3_), 4.16 (2H, q, J = 4, CH_2_), 6.89–8.22 (8H, m, Ar-H), 8.31 (1H, s, R-NHC(=O)NH-C_6_H_5_-NO_2_), 10.13 (1H, s, R-NHC(=O)NH-C_6_H_5_-NO_2_). ^13^C NMR (100.6 MHz, DMSO) δ ppm 15.17, 64.53, 112.40, 117.77, 119.32, 120.98, 123.10, 125.70, 128.58, 141.44, 146.90, 147.53, 152.27. *m*/*z* = 302.6 (M^+^ +1), 137.29, 301.63, 111.86, 97.04, 73.38, 86.51. IR (ATR) υ_max_ (cm^−1^): 741.66, 1110.03, 1321.61, 1499.36, 1530.14, 1706.16, 2920.36, 3357.46. HRMS-HESI: [M+Na]^+^ calculated = 302.11353; observed = 302.11415.

#### 3.2.5. Synthesis of Compounds **11**–**12**

4′-isobutylacetophenone (Scheme 5—compound A for synthesis of compound **11**) or 2-acetyl-6-methoxynaphthalene (Scheme 5—compound A for synthesis of compound **12**) (17 mmol, 1 eq) and hydroxylamine hydrochloride (35 mmol, 2.06 eq) were dissolved in a mixture of ethanol (25 mL) and pyridine (25 mL) and heated at 50 °C for 2 h. Most of the solvent was removed in vacuo, and the residue was dissolved in ether and washed with 2N HCl (50 mL). The solution was dried over MgSO_4_ and evaporated to give compound B (Scheme 5).

The oxim (compound B, Scheme 5) (5 mmol, 1 eq) was dissolved in ethanol (10 mL) and cooled to 0 °C. The borane–pyridine complex (15 mmol, 3 eq) was added via syringe under nitrogen, and then 6 M HCl (5 mL) 10 min later. The reaction was completed within 30 min and the pH was adjusted to 9 with 2M NaOH. The mixture was extracted using ethyl acetate and dried over MgSO_4_ to give compound C (Scheme 5).

Acetyl chloride (5.73 mmol, 2.2 eq) was added to a solution of TEA (7.8 mmol, 3 eq) and compound C (2.6 mmol, 1 eq) in DCM (10 mL) at 0 °C. After stirring for 30 min, the mixture was added to 2M HCl (10 mL). The organic layer was dried over MgSO_4_ and evaporated to give compound D (Scheme 5).

Compound D (0.36 mmol, 1 eq) was dissolved in 2-propanol (1 mL) and a solution of lithium hydroxide (207 mg/mL) in water (0.5 mL) was added. The mixture was stirred for 30 min and the compound was extracted using 2 M HCl (5 mL) and ether (10 mL). The organic layer was dried and evaporated. The product was further purified by preparative TLC using ethyl acetate–cyclohexane 15:1 *v*/*v* to give compounds **11** and **12**.

*N*-hydroxy-*N*-[1-(6-isobutyl-naphthalen-2-yl)-ethyl]-acetamide (**11**). Yellow crystalline solid. Yield: 33%. Melting point: 96.5–98 °C. 1H NMR (400 MHz, DMSO) δ ppm 0.85 (6H, d, J = 6.4, (CH3)_2_CH-R), 1.43 (3H, d, J = 7.2, CH_3_), 1.77–1.84 (1H, m, (CH3)2CH-R), 2.00 (3H, s, C(=O)CH3), 2.41 (2H, d, J = 7.2, CH2), 5.58 (1H, s, (OH)N-CH-R), 7.09–7.22 (4H, m, Ar-H), 9.51 (1H, s,OH). 13C NMR (100.6 MHz, DMSO-d6) δ 17.62, 21.19, 22.64, 30.06, 44.71, 52.75, 127.26, 129.14, 139.06, 140.22, 170.74. *m*/*z* = 236.2 (M+ +1), 161.28, 119.31, 117.30, 91.32, 105.31, 115.30. IR (ATR) υ_max_ (cm^−1^): 739.74, 845.13, 985.65, 1122.97, 1201.08, 1419.62, 1458.62, 1592.93, 2161.77, 2867.72, 2954.32, 3152.59. HRMS-HESI: [M+Na]+ calculated = 258.14645; observed = 258.14605.

*N*-hydroxy-N-[1-(6-methoxy-naphthalen-2-yl)-ethyl]-acetamide (**12**). White crystalline solid. Yield: 16%. Melting point: 168–170 °C. ^1^H NMR (400 MHz, DMSO) δ ppm 1.52 (3H, d, J = 6.8, R-CH(CH_3_)-R1), 2.02 (3H, s, C(=O)CH_3_), 3.85 (3H, s, ROCH_3_), 5.74 (1H, d, J = 4, R-CH(CH_3_)-R1), 7.12–7.81 (6H, m, Ar-H), 9.59 (1H, s, OH). ^13^C NMR (100.6 MHz, DMSO-d6) δ 17.44, 21.22, 52.99, 55.60, 106.10, 119.01, 125.69, 126.70, 127.04, 128.62, 129.78, 133.89, 136.85, 157.62, 170.83. *m*/*z* = 260.2 (M^+^ +1), 185.20, 170.20, 141.25, 153.22, 152.21, 169.20. IR (ATR) υ_max_ (cm^−1^): 693.29, 756.36, 820.06, 853.42, 948.01, 1027.56, 1163.47, 1181.92, 1452.17, 1605.90, 2788.64, 2985.80. HRMS-HESI: [M+Na]^+^ calculated = 282.11006; observed = 282.10977.

#### 3.2.6. Synthesis of Compound **13**

A suspension of 3,5-di-tert-butyl-4-hydroxybenzaldehyde (0.007 mol), 3-aminorhodanine (0.0065 mol), and sodium acetate (0.02325 mol) in acetic acid (10 mL) was stirred at reflux for 48 h. The cooled mixture was added to water (about 200 mL) and the precipitate was washed with water and purified by preparative TLC using dichloromethane to give compound **13**.

3-amino-5-(3,5-di-tert-butyl-4-hydroxy-benzylidene)-2-thioxo-thiazolidin-4-one (13). Orange crystalline solid. Yield: 25%. Melting point: 244.5–246.5 °C. 1H NMR (400 MHz, DMSO) δ ppm 1.42 (18H, s, 2 × (C(CH3)3), 5.96 (2H, s, NH2), 7.42 (2H, s, Ar-H), 7.84 (1H, s, R=CH-Ar), 7.97 (1H, s, OH). 13C NMR (100.6 MHz, DMSO-d6) δ 30.39, 35.07, 115.97, 124.87, 128.95, 135.78, 140.03, 157.95, 164.14, 187.39. *m*/*z* = 364.9 (M+ +1), 308.97, 252.91, 176.95, 150.00, 57.32, 233.00. IR (ATR) υmax (cm^−1^): 3607.02, 3321.78, 2949.73, 1709.09, 1580.24, 1417.54, 1112.15, 898.91, 723.28. HRMS-HESI: [M+H]+ calculated = 365.13520; observed = 365.13559.

### 3.3. In Vitro COX-1, COX-2 and 5-LOX Inhibition Assay

#### 3.3.1. COX-1 and COX-2 Inhibition

The COX-1 and COX-2 inhibitory activates of the synthesized compounds were tested using fluorometric COX-1 (Catalog Number: ab204698) and COX-2 (Catalog Number: ab283401) inhibitor screening kits (Abcam, United Kingdom). These assays are based on the fluorometric detection of Prostaglandin G2, generated by the COX enzyme. The experiment was conducted according to the manufacturer’s instructions. Each assay consists of a COX Assay Buffer, COX Probe (in DMSO), COX Cofactor (in DMSO), arachidonic acid and NaOH. The COX-1 inhibitor screening kit contains COX-1 ovine enzyme and COX-1 inhibitor SC560 (dissolved in DMSO), while the COX-2 inhibitor screening kit contains COX-2 human recombinant enzyme and COX-2 inhibitor Celecoxib (dissolved in DMSO). The tested compounds were dissolved in DMSO and further diluted five times with the COX Assay Buffer. The Inhibitor Control (IC) was prepared by adding 2 µL of COX-1/COX-2 inhibitor and 8 µL of COX Assay Buffer into assigned wells, while the Solvent Control (SC) was prepared by adding 2 µL of DMSO and 8 µL of COX Assay Buffer into assigned wells and the Sample (S) and Enzyme Control (EC) were prepared by adding 10 µL of diluted tested compounds or COX Assay Buffer into assigned wells. The Reaction Master Mix (COX Assay Buffer, COX Probe, Diluted COX Cofactor and COX-1/COX-2 Enzyme) was prepared according to instructions and 80 µL of the Reaction Master Mix was added into each well. Meanwhile, a diluted arachidonic acid/NaOH solution was prepared and 10 µL was added into each well using a multi-channel pipette to initiate all the reactions at the same time. The fluorescence values (Ex/Em = 535/587 nm) of the samples were kinetically measured at 25 °C for 5–10 min. The EC, SC, IC and tested compounds at all concentrations were analyzed in triplicates. Two points (T_1_ and T_2_) in the linear range of the plot were chosen and corresponding fluorescence values (RFU_1_ and RFU_2_) were obtained. The slope for all samples was calculated by dividing ΔRFU (RFU_2_-RFU_1_) by time Δt (T_2_−T_1_). The percentage of inhibition was calculated as follows:

$$\%\;Relative\;Inhibition=\frac{Slope\;of\;EC-Slope\;of\;S}{Slope\;of\;EC}*100$$

Fluorescence measurements were performed on a BioTek Synergy LX multi-mode reader (Winooski, VT, USA).

#### 3.3.2. 5-LOX Inhibition

The 5-LOX inhibitory activates of synthesized compounds were tested using a fluorometric 5-LOX inhibitor screening kit (MyBioSource, San Diego, CA, USA; Catalog Number: MBS846911). This assay is based on the fluorometric detection of the intermediate generated by the 5-LOX enzyme. The experiment was conducted according to the manufacturer’s instructions. The 5-LOX kit consists of the LOX Assay Buffer, LOX Probe, LOX Substrate, 5-LOX inhibitor Zileuton and 5-LOX Enzyme. The tested compounds were dissolved in DMSO. Test solutions were added to corresponding wells at an amount of 2 µL out of the final 100 µL reaction volume per well. The Enzyme Control (EC), Solvent Control (SC) and Inhibitor Control (IC) were prepared by adding 2 µL of Assay Buffer, DMSO and Zileuton into each well, respectively. The volume was adjusted to 40 µL in each well by adding 38 µL of LOX Assay Buffer. The Reaction Mix (containing LOX Assay Buffer, LOX Probe and 5-LOX Enzyme) was prepared according to the instructions and 40 µL of the Reaction Mix was added into each well. The plate was incubated at room temperature for 10 min before adding the substrate diluted in the LOX Assay Buffer (1:2500 dilution factor). The final substrate solution was added at an amount of 20 µL into each well using a multi-channel pipette to initiate all the reactions at the same time. The fluorescence values (Ex/Em = 500/536 nm) of the samples were kinetically measured in the second minute after the addition of the substrate at 30 s intervals for 10–20 min. The EC, SC, IC and tested compounds at all concentrations were analyzed in triplicates. Two points (T1 and T2) in the linear range of the plot were chosen and corresponding fluorescence values (RFU1 and RFU2) were obtained. The slope for all samples was calculated by dividing ΔRFU (RFU2-RFU1) by the time Δt (T_2_−T_1_). The percentage of inhibition was calculated according to the equation mentioned in Section 3.3.1.

Fluorescence measurements were performed on a BioTek Synergy LX multi-mode reader (Winooski, VT, USA).

### 3.4. Evaluation of Antioxidant Activity of Synthesized Compounds

#### 3.4.1. Sample Collection

The remains of the serum samples were taken from healthy subjects after biochemical analysis; these subjects participated in regular medical examinations at the Military Medical Academy in Belgrade and consented to the formation of a serum pool. Neither the patients’ data nor their identities were used. Serum pool samples were prepared and aliquoted (450 µL portions) according to the routine procedure for in-house controle samples and preserved at the -80 °C until analysis. Five serial dilutions of the tested compounds (concentrations: 1000 µM, 500 µM, 250 µM, 125 µM and 62.5 µM) were added alone (50 µL) or in combination (25 µL) with oxidant TBH (25 µM) into 450 µL of serum. The compounds were dissolved in dimethyl sulfoxide (DMSO) for stock solution (1000 µM), while a PBS buffer (phosphate buffered saline) pH = 7.4 was used for serial dilutions (500 µM, 250 µM, 125 µM and 62.5 µM). Deionized water was used as a blank. DMSO and buffer pH = 7.4 were used as a control, while trolox solution (2 mmol/L) was used as a standard. All samples were incubated in duplicate for 2 h and 24 h at 37 °C.

#### 3.4.2. Biochemical Parameters

Oxidative stress parameters (total antioxidative status (TAS), total oxidative status (TOS), total concentration of sulfhydryl group (SHG) and prooxidative–antioxidative balance (PAB)) were determined in order to calculate prooxidative score, antioxidative score and oxy score (OS). These parameters were determined in serum.

Total antioxidative status (TAS) was measured using Erel’s method [34], which was further optimized by Kotur-Stevuljevic et al. in 2015 [34]. ABTS (2,2-azino-bis(3-ethyl-benzthiazoline-6-sulfonic acid) is oxidized to a green-colored ABTS^+^ cation by hydrogen peroxide in the acetate buffer (pH = 3.6). This stable cation is chromogen, and the presence of antioxidants in the samples causes its discoloration proportional to their concentrations. The absorbance was measured using a SPECTROstar Nano Microplate Reader (BMG Labtech, Ortenberg, Germany). The reaction was calibrated with Trolox and the TAS values are expressed as mmol trolox equivalent/L.

Total oxidative status (TOS) was measured using Erel’s method [35,36]. Here, a ferrous ion-*o*-dianisidine complex is oxidized to ferric ion by the oxidants present in the sample. The ferric ion forms a colored complex with xylenol orange in an acidic environment and in the presence of glycerol. The intensity of the color of the formed complex is proportional to the total contents of oxidants in the sample. The absorbance was measured at 560 nm using a SPECTROstar Nano Microplate Reader (BMG Labtech, Ortenberg, Germany). The reaction was calibrated with hydrogen peroxide and the TOS values are expressed as µmol H_2_O_2_ equivalent/L.

Prooxidant–antioxidant balance (PAB) was measured according to procedures in the literature [35,37]. Here, hydrogen peroxide is determined in the presence of antioxidants in the sample. Both of them (hydrogen peroxide and antioxidants (including uric acid)) react with chromogen TMB (3,3′,5,5′-tetramethylbenzidine) simultaneously. The hydrogen peroxide and chromogen reaction is enzymatically catalyzed with peroxidase, while the antioxidants–chromogen reaction is non-enzymatic. The intense blue color, produced as a result of the reaction between hydrogen peroxide and TMB, is decolorized by the reaction of the antioxidant with the TMB. The resulting color (result of two competitive reactions) is proportional to the ratio of antioxidants to prooxidants. The absorbance was measured at 450 nm. Different ratios of hydrogen peroxide and uric acid solutions were used to make a standard curve. The PAB results are expressed in hydrogen peroxide concentrations.

Total Sulfydryl Groups Content (SHG) was measured using a modified version of Ellman’s method [35,38]. This method is based on the reaction between DTNB (2,2′-dinitro-5,5′-dithiobenzoic acid) and aliphatic thiol compounds in an alkaline environment (pH = 9.0). As a result of the reaction, 1 mol of yellow-colored p-nitrophenol anion per mol of thiol is produced, and the absorbance was measured at 412 nm.

Oxy score (OS) represents the difference between prooxidative score (average value of Z scores of all measured prooxidants, i.e., TOS and PAB) and antioxidative score (average value of Z scores of all measured antioxidants, i.e., TAS and SHG). Z score is the difference between the original value and the control value divided by the SD of the control values. Control values are derived from the prooxidant and antioxidant values in the native serum pool. A lower OS value indicates stronger antioxidative protection.

#### 3.4.3. Statistics

The statistical analysis was performed using SPSS 18.0 (SPSS, INC. Chicago, IL, USA). OS values are presented as medians ± (interquartile range). In order to compare and determine whether there were statistically significant differences between samples with and without TBH, Wilcoxon’s paired test was performed. Wilcoxon’s paired test was used in order to check whether there were statistically significant differences between 2 h and 24 h incubation.

## 4. Conclusions

According to the literature overview, thirteen compounds with potential dual COX-2 and 5-LOX inhibitory activities were synthesized and characterized. The in vitro evaluation of COX-2 and 5-LOX inhibitory activity was performed using fluorometric screening kits. Seven compounds (**1**, **2**, **3**, **5**, **6**, **11** and **12**) showed dual COX-2 and 5-LOX inhibitory activities. In addition, these compounds showed weak COX-1 inhibitory activities, i.e., good COX-2/COX-1 selectivity. The introduction of a N-hydroxyurea group into the NSAIDs (componds **1**, **2** and **3**) leads to the manifestation of a 5-LOX inhibitory activity, while the COX-2 inhibitory effect is preserved (COX-2 inhibition is higher if the starting NSAID is a stronger COX-2 inhibitor). Of the 3,5-di-tert-butylphenol derivatives, compound **5** was singled out, and it can be assumed that good dual inhibitory activity is obtained by the introduction of short-carbon chain amino acids. Small structural changes in the heterocyclic ring of the darbufelone-like derivative (**13**) did not lead to the manifestation of dual inhibition. Both of the “type B hydroxamic acids” derivatives (**11** and **12**) proved to be dual inhibitors. The two most potent dual COX-2 and 5-LOX inhibitors were compounds **2** and **3,** with IC_50_ values < 10 µM (COX-2) and < 2 µM (5-LOX). The majority of the synthesized compounds showed antioxidant properties, including dual COX-2 and 5-LOX inhibitors **1**, **3**, **5**, **11** and **12**. Since chronic inflammation is thought to cause oxidative stress and cancer, it is important that compounds possess antioxidant properties in addition to anti-inflammatory effects (COX-2 and 5-LOX inhibition).

## Figures and Tables

**Table 1:** In vitro COX-1, COX-2 and 5-LOX enzymes’ inhibition results for compounds **1**–**13**, celecoxib and zileuton.

Compound	COX-2 IC_50_ (µM)	5-LOX IC_50_ (µM)	COX-1 IC_50_ (µM)
**1**	18.28 ± 2.17	5.71 ± 0.15	>100
**2**	6.72 ± 0.79	1.62 ± 0.67	>100
**3**	5.26 ± 0.34	1.73 ± 0.64	>100
**4**	>100	8.10 ± 2.12	Not tested
**5**	10.64 ± 0.80	9.30 ± 1.87	>100
**6**	6.89 ± 0.83	53.84 ± 11.87	>100
**7**	>100	12.55 ± 3.36	Not tested
**8**	>100	14.52 ± 3.62	Not tested
**9**	>100	>100	Not tested
**10**	>100	13.01 ± 2.67	Not tested
**11**	36.18 ± 3.08	1.04 ± 0.22	>100
**12**	83.42 ± 4.37	1.29 ± 0.10	>100
**13**	>100	13.58 ± 3.54	Not tested
Celecoxib	0.07 ± 0.01	Not tested	Not tested
Zileuton	Not tested	0.36 ± 0.10	Not tested

**Table 2:** Oxy scores (OS) obtained for tested compounds **1**–**13**, BHT, urea, celecoxib and zileuton after 2 h and 24 h incubation, before and after addition of TBH.

	2 h Incubation				24 h Incubation	
		TBH added	*p*		TBH added	*p*
1	−0.43 (−1.27–3.13)	6.44 (5.51–7.02)	0.023	0.71 (0.51–0.80)	5.46 (4.96–6.20)	<0.001
2	3.28 (−0.68–16.66)	5.20 (3.70–5.42)	0.597	0.33 (−0.07–0.85)	6.79 (4.37–7.90)	<0.001
3	1.58 (−0.38–3.45)	5.81 (4.64–7.25)	0.023	1.31 (−0.31–1.75)	1.93 (0.65–5.02) ^#^	0.226
4	0.24 (−0.24–0.89)	3.68 (3.36–8.40)	0.001	3.00 (1.99–3.93) **	7.86 (7.20–10.68)	0.016
5	0.29 (−0.30–1.23)	2.83 (2.55–3.39)	<0.001	2.14 (1.37–2.62) **	5.64 (5.07–6.47)	<0.001
6	0.52 (0.08–1.52)	3.77 (3.37–4.20)	0.002	3.09 (2.50–3.67)	6.07 (4.34–7.02)	0.016
7	3.12 (2.21–3.47)	6.62 (6.42–6.98)	0.021	2.55 (2.18–2.92)	10.54 (4.45–27.42)	0.021
8	0.03 (−0.96–1.46)	8.43 (5.76–13.81)	0.010	−0.52 (−1.24–3.03)	−0.55 (−0.67–1.48) ^##^	0.762
9	0.09 (−0.79–1.80)	5.97 (4.06–8.25)	0.003	−0.18 (−1.55–2.91)	−0.19 (−0.68–1.56)	0.450
10	−0.64 (−0.84−0.30)	6.34 (5.07–22.02)	<0.001	−0.97 (−1.53–3.85)	0.97 (−0.04–4.08)	0.199
11	−0.22 (−0.65–0.82)	5.15 (4.76–6.74)	<0.001	1.83 (1.49–2.18) *	3.18 (2.92–3.60) ^##^	<0.001
12	0.24 (−0.06–0.58)	4.79 (4.25–5.43)	<0.001	2.49 (2.03–2.66) **	3.97 (3.44–6.33)	<0.001
13	0.75 (0.56–0.94)	5.89 (5.63–6.14)	<0.001	0.64 (0.19–1.01)	2.35 (2.10–3.92) ^#^	<0.001
BHT	3.08 (2.73–3.26)	7.98 (6.77–8.81)	0.021	2.62 (2.14–3.22)	4.51 (4.38–7.22)	0.021
Celecoxib	5.66 (2.92–12.90)	7.94 (6.97–8.45)	0.564	2.77 (2.46–3.27)	4.95 (4.57–10.25)	0.021
Zileuton	2.60 (−7.86–7.54)	6.87 (6.52–7.18)	0.248	3.61 (2.47–4.93)	8.80 (5.71–13.66)	0.043
Urea	3.48 (3.44–4.08)	8.11 (7.61–8.79)	0.021	4.60 (3.62–6.02)	5.19 (4.96–14.00)	0.149
Trolox	0.54 (−0.66–2.43)	/	/	0.07 (−0.59–0.59) *	/	/
TBH	6.40 (4.66–11.59)	/	/	6.43 (4.73–12.00)	/	/

Results of Wilcoxon’s paired tests; statistically significant differences in samples before and after TBH addition are presented as *p* values (<0.05); statistically significant differences between 2 h and 24 h incubation are marked as follows—24 h vs. 2 h: * *p* < 0.05, ** *p* < 0.01; 24 h TBH vs. 2 h TBH: ^#^ *p* < 0.05, ^##^ *p* < 0.01.

## Data Availability

Data is contained within the article and supplementary material.

## Supplementary Materials

The following supporting information can be downloaded at: https://www.mdpi.com/article/10.3390/ph16040549/s1, Figure S1: ^1^H NMR spectrum (a) and ^13^C NMR (b) of compound **1** (**IND-NHU**); Figure S2: ^1^H NMR spectrum (a) and ^13^C NMR (b) of compound **2** (**FLU-NHU**).Table S1: title; Figure S3: ^1^H NMR spectrum (a) and ^13^C NMR (b) of compound **3** (**DIKLO-NHU**); Figure S4: ^1^H NMR spectrum (a) and ^13^C NMR (b) of compound **4** (**BHTK-MHIS**); Figure S5: ^1^H NMR spectrum (a) and ^13^C NMR (b) of compound **5** (**BHTK-MGLY**); Figure S6: ^1^H NMR spectrum (a) and ^13^C NMR (b) of compound **6** (**BHTK-AA**); Figure S7: ^1^H NMR spectrum (a) and ^13^C NMR (b) of compound **7** (**BHTK-PA**); Figure S8: ^1^H NMR spectrum (a) and ^13^C NMR (b) of compound **8** (**2A**); Figure S9: ^1^H NMR spectrum (a) and ^13^C NMR (b) of compound **9** (**2D**); Figure S10: ^1^H NMR spectrum (a) and ^13^C NMR (b) of compound **10** (**QSAR17**); Figure S11: ^1^H NMR spectrum (a) and ^13^C NMR (b) of compound **11** (**IBU-Ac**); Figure S12: ^1^H NMR spectrum (a) and ^13^C NMR (b) of compound **12** (**NAP-Ac**); Figure S13: ^1^H NMR spectrum (a) and ^13^C NMR (b) of compound **13** (**BHTA-3AR**); Tables S1–S4: Statistically significant differences (Wilcoxon test) between tested compounds.
