# Supplementary material for: Synthesis, Evaluation of Enzyme Inhibition and Redox Properties of Potential Dual COX-2 and 5-LOX Inhibitors
Source: Pharmaceuticals (Basel). 2023 Apr 6;16(4):549. doi: 10.3390/ph16040549 (PMC10142505; doi:10.3390/ph16040549)
Supplement: Supplementary file 1 [file pharmaceuticals-16-00549-s001.zip › pharmaceuticals-2288881-supplementary.pdf]

Supplementary Information associated with the paper

Contents

1. NMR spectra of compounds 1–13.

^{1}H NMR spectrum (a) and ^{13}C NMR (b) of compound 1 (IND-NHU). 3

^{1}H NMR spectrum (a) and ^{13}C NMR (b) of compound 2 (FLU-NHU). 4

^{1}H NMR spectrum (a) and ^{13}C NMR (b) of compound 3 (DIKLO-NHU). 5

^{1}H NMR spectrum (a) and ^{13}C NMR (b) of compound 4 (BHTK-MHIS). 6

^{1}H NMR spectrum (a) and ^{13}C NMR (b) of compound 5 (BHTK-MGLY). 7

^{1}H NMR spectrum (a) and ^{13}C NMR (b) of compound 6 (BHTK-AA). 8

^{1}H NMR spectrum (a) and ^{13}C NMR (b) of compound 7 (BHTK-PA). 9

^{1}H NMR spectrum (a) and ^{13}C NMR (b) of compound 8 (2A). 10

^{1}H NMR spectrum (a) and ^{13}C NMR (b) of compound 9 (2D). 11

^{1}H NMR spectrum (a) and ^{13}C NMR (b) of compound 10 (QSAR17). 12

^{1}H NMR spectrum (a) and ^{13}C NMR (b) of compound 11 (IBU-Ac). 13

^{1}H NMR spectrum (a) and ^{13}C NMR (b) of compound 12 (NAP-Ac). 14

^{1}H NMR spectrum (a) and ^{13}C NMR (b) of compound 13 (BHTA-3AR). 15

2. Statistically significant differences (Wilcoxon test) between tested compounds. 16-17

(a)

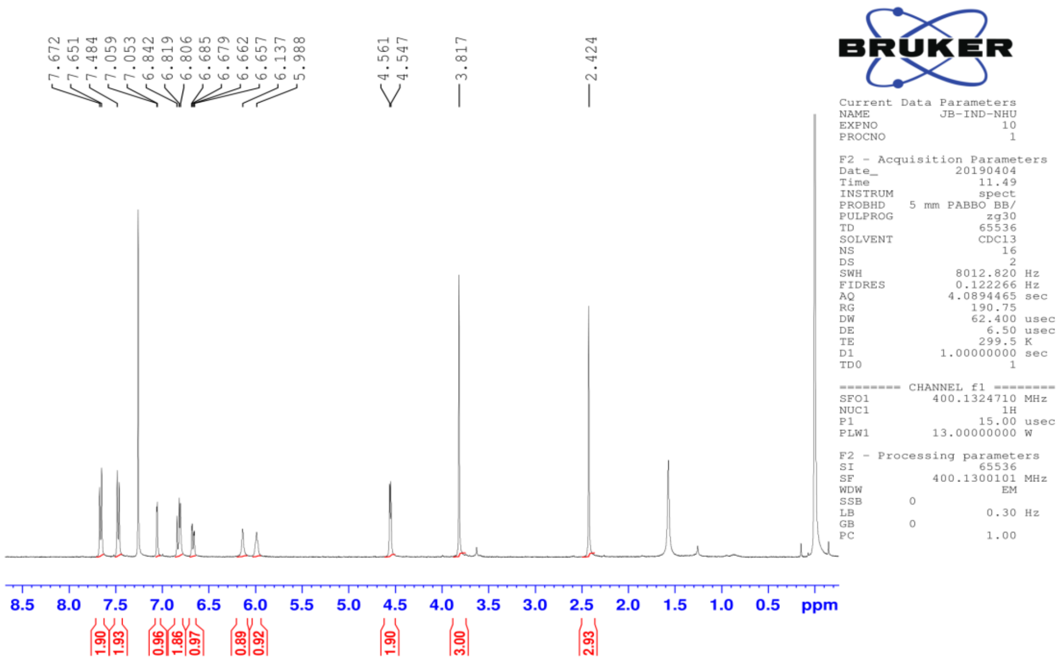
7.672
7.651
7.484
7.059
7.053
6.842
6.819
6.806
6.685
6.679
6.662
6.657
6.137
5.988
4.561
4.547
3.817
2.424
BRUKER
Current Data Parameters
NAME JB-IND-NHU
EXPNO 10
PROCNO 1
F2 - Acquisition Parameters
Date_ 20190404
Time 11.49
INSTRUM spect
PROBHD 5 mm PABBO BB/
PULPROG zg30
TD 65536
SOLVENT CDCl3
NS 16
DS 2
SWH 8012.820 Hz
FIDRES 0.122266 Hz
AQ 4.0894465 sec
RG 190.75
DW 62.400 usec
DE 6.50 usec
TE 299.5 K
D1 1.00000000 sec
TD0 1
======== CHANNEL f1 ========
SFO1 400.1324710 MHz
NUC1 1H
P1 15.00 usec
PLW1 13.00000000 W
F2 - Processing parameters
SI 65536
SF 400.1300101 MHz
WDW EM
SSB 0
LB 0.30 Hz
GB 0
PC 1.00
8.5 8.0 7.5 7.0 6.5 6.0 5.5 5.0 4.5 4.0 3.5 3.0 2.5 2.0 1.5 1.0 0.5 ppm
1.90
1.93
0.96
1.86
0.97
0.89
0.92
1.90
3.00
2.93

(b)

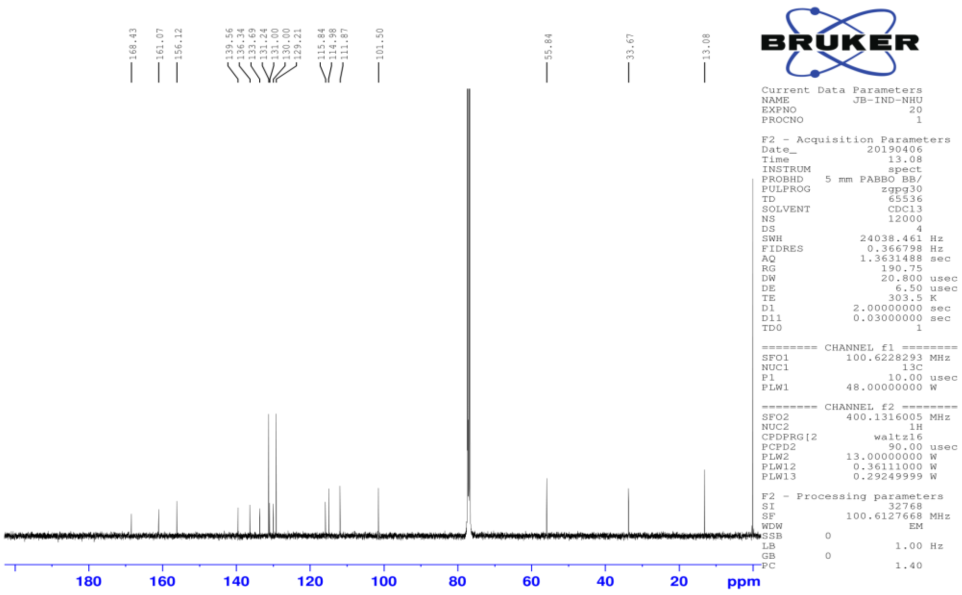

Figure S1. ^{1}H NMR spectrum (**a**) and ^{13}C NMR (**b**) of compound **1** (**IND-NHU**).

(a)

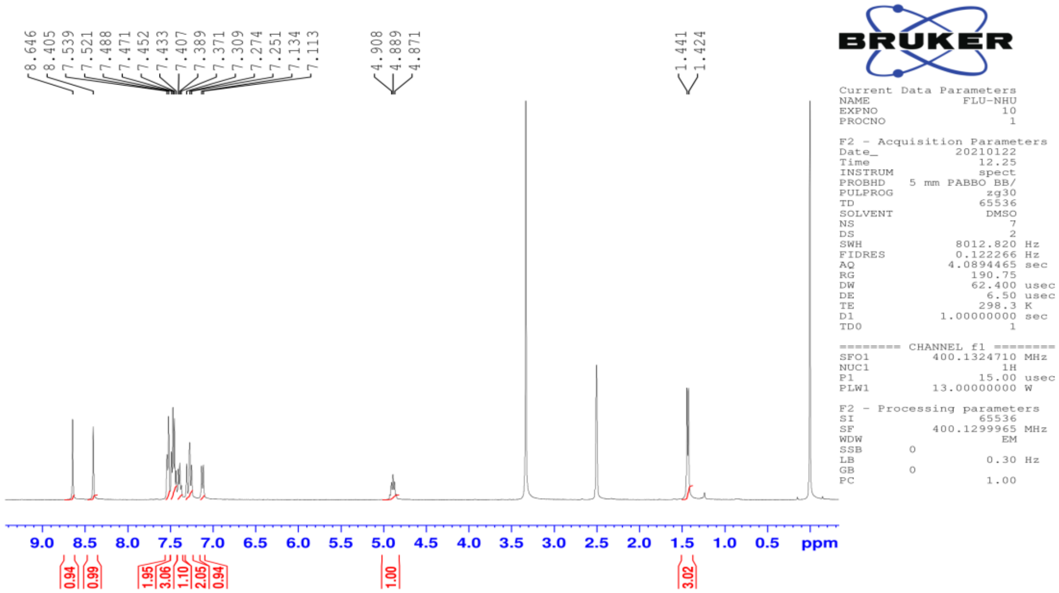

(b)

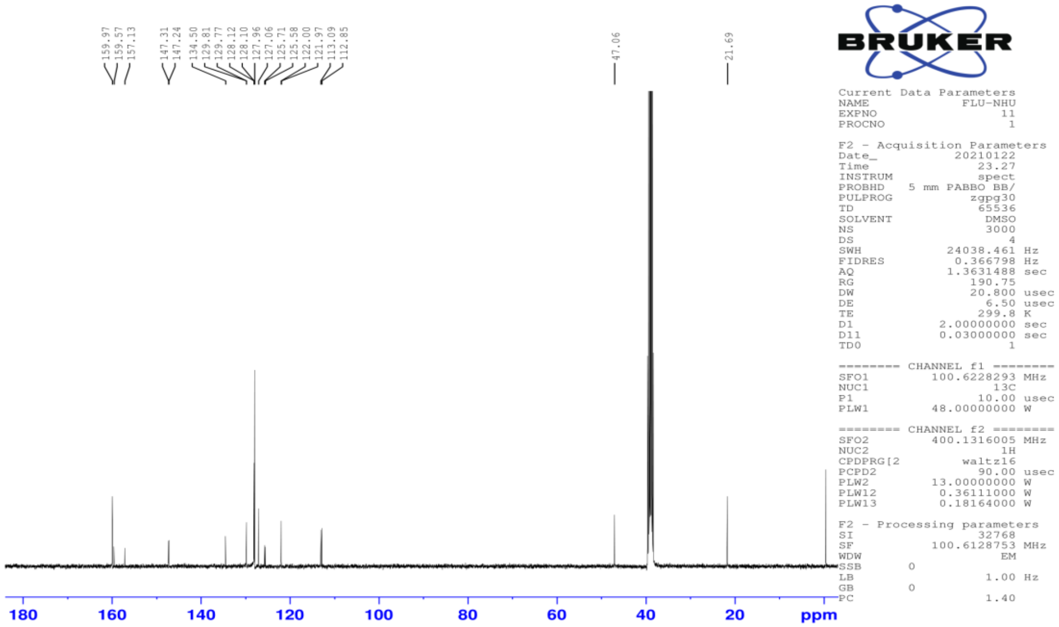

Figure S2. ^{1}H NMR spectrum (**a**) and ^{13}C NMR (**b**) of compound **2** (**FLU-NHU**).

(a)

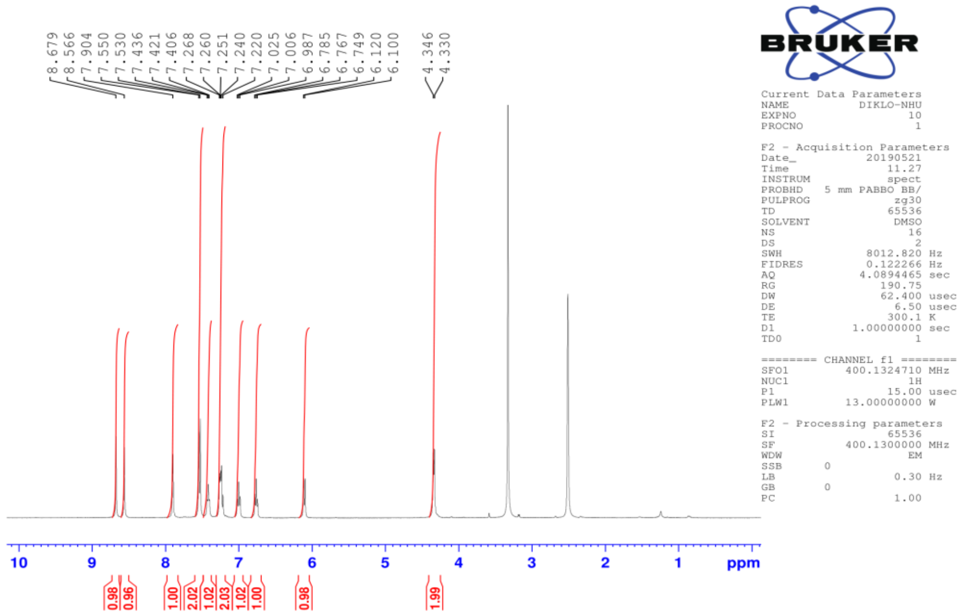

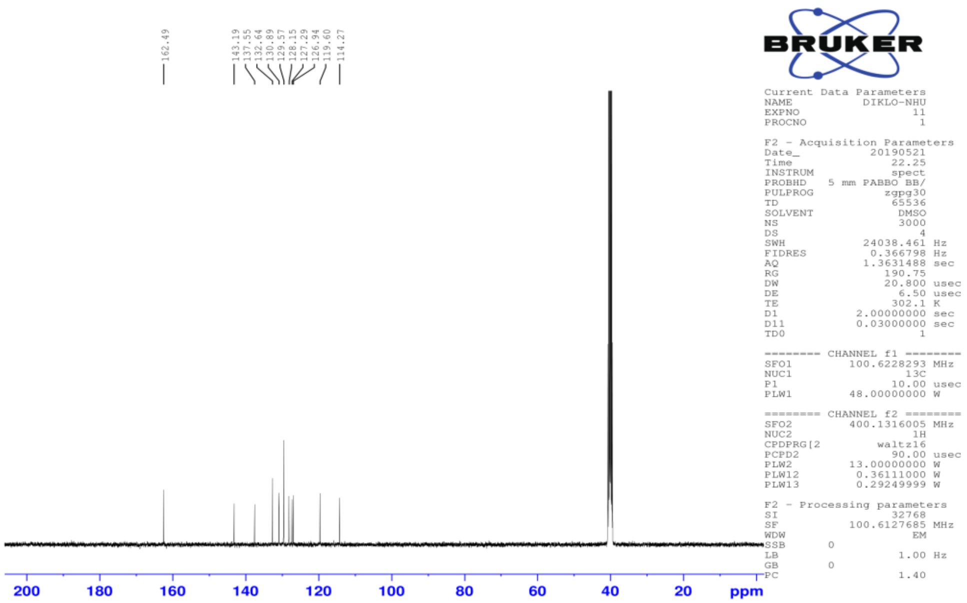

(b)

Figure S3. ^{1}H NMR spectrum (**a**) and ^{13}C NMR (**b**) of compound **3** (**DIKLO-NHU**).

(a)

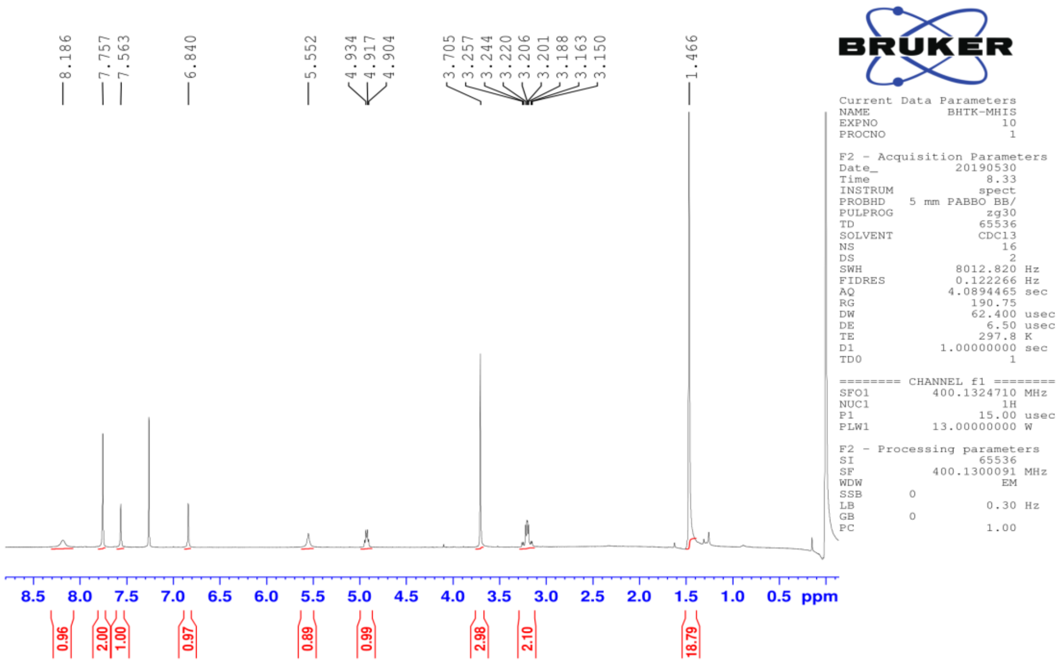

(b)

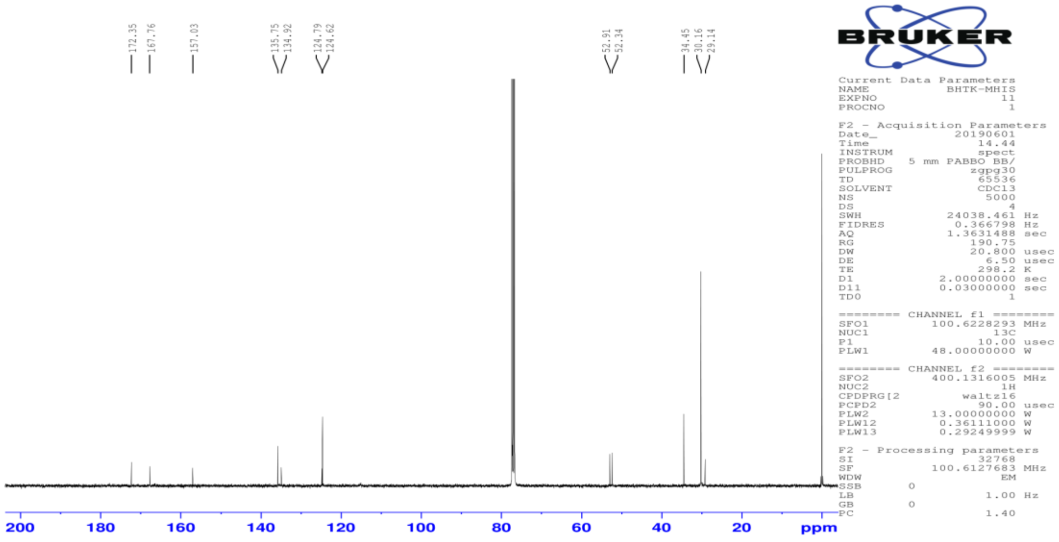

Figure S4. ^{1}H NMR spectrum (**a**) and ^{13}C NMR (**b**) of compound **4** (**BHTK-MHIS**).

(a)

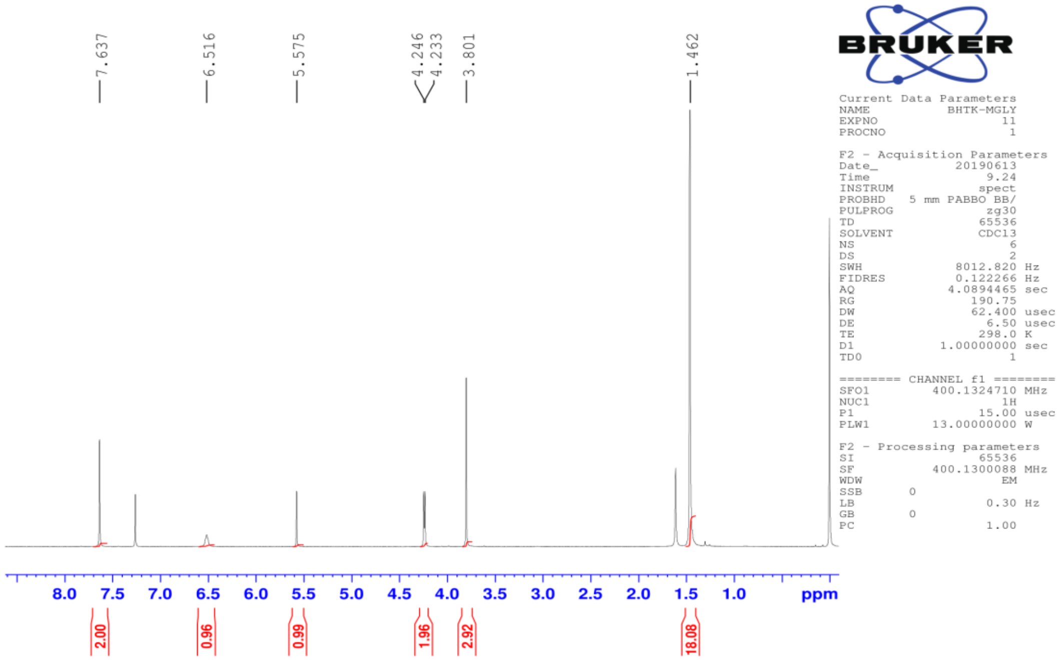

(b)

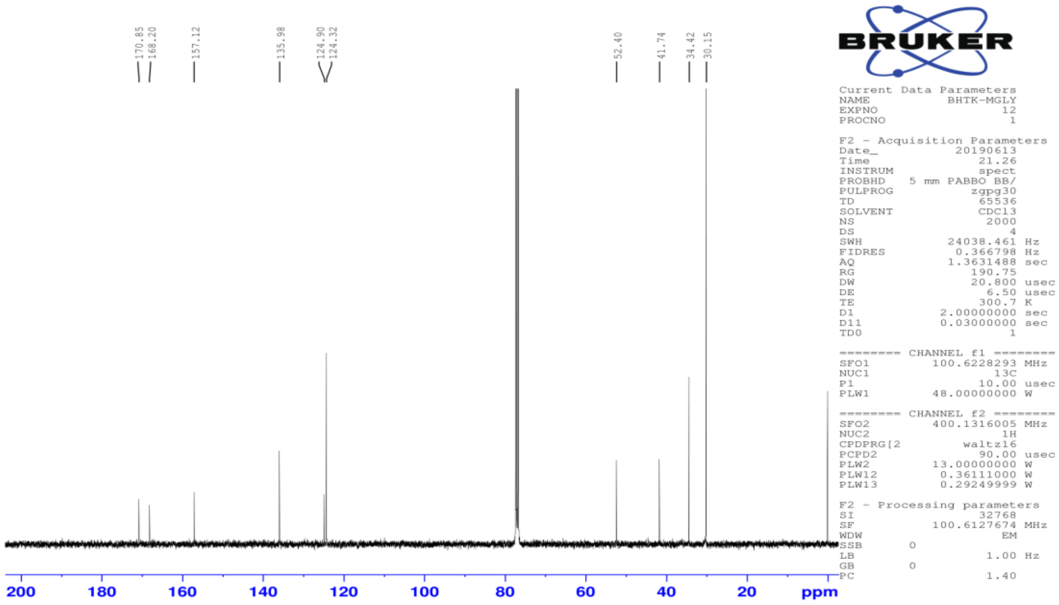

Figure S5. ^{1}H NMR spectrum (**a**) and ^{13}C NMR (**b**) of compound **5** (**BHTK-MGLY**).

(a)

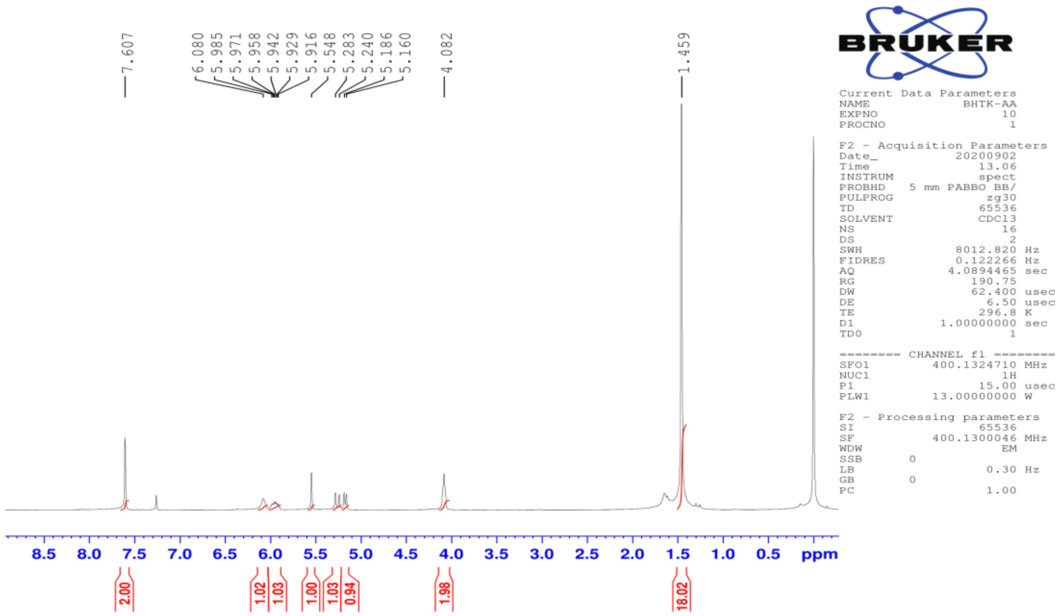

(b)

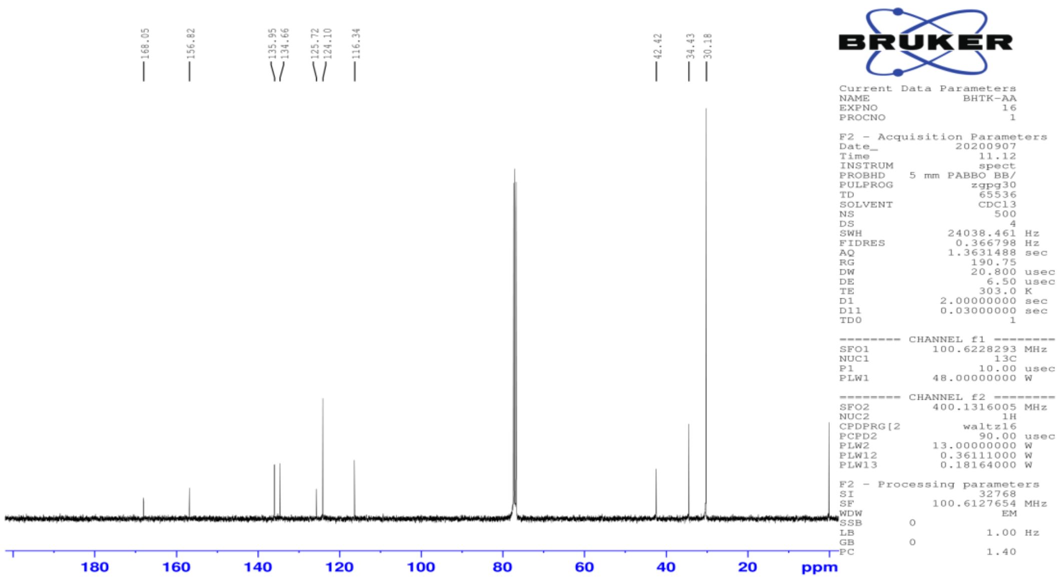

Figure S6. ^{1}H NMR spectrum (**a**) and ^{13}C NMR (**b**) of compound **6** (**BHTK-AA**).

(a)

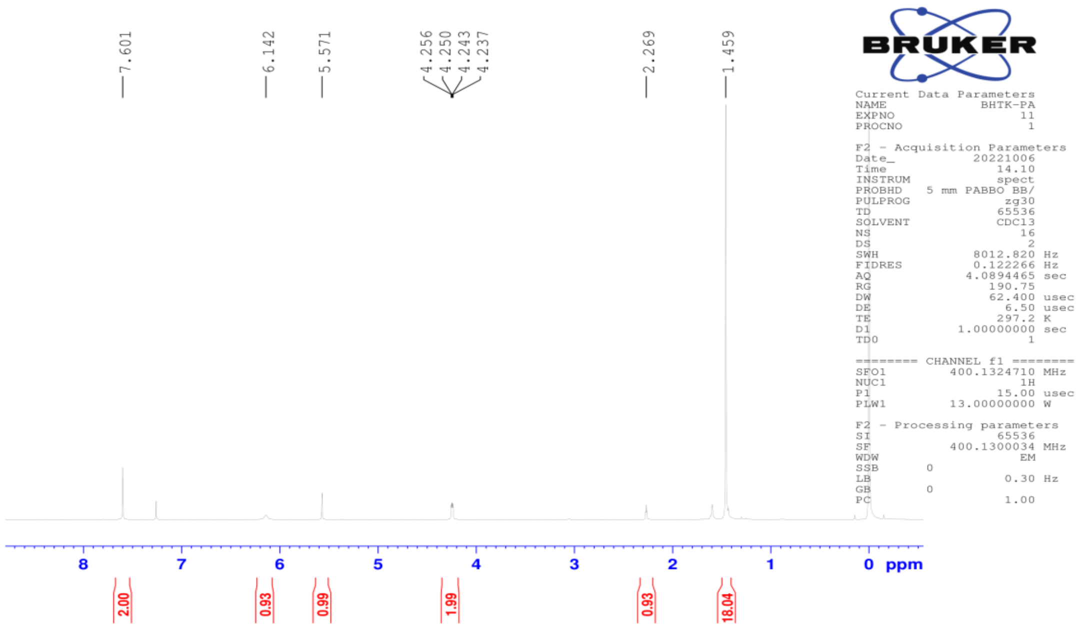

(b)

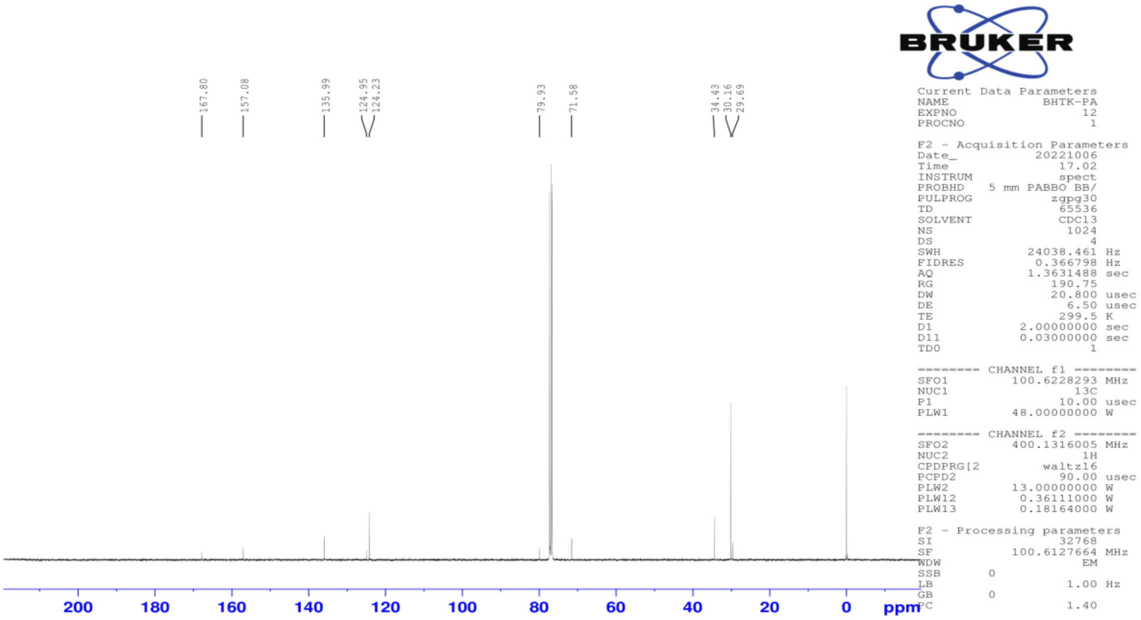

Figure S7. ^{1}H NMR spectrum (**a**) and ^{13}C NMR (**b**) of compound **7** (**BHTK-PA**).

(a)

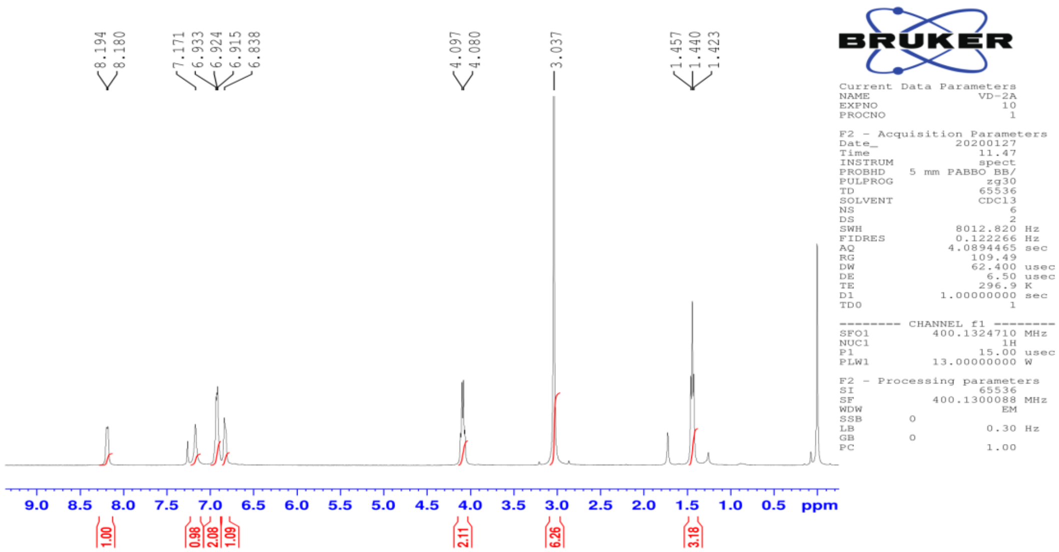

(b)

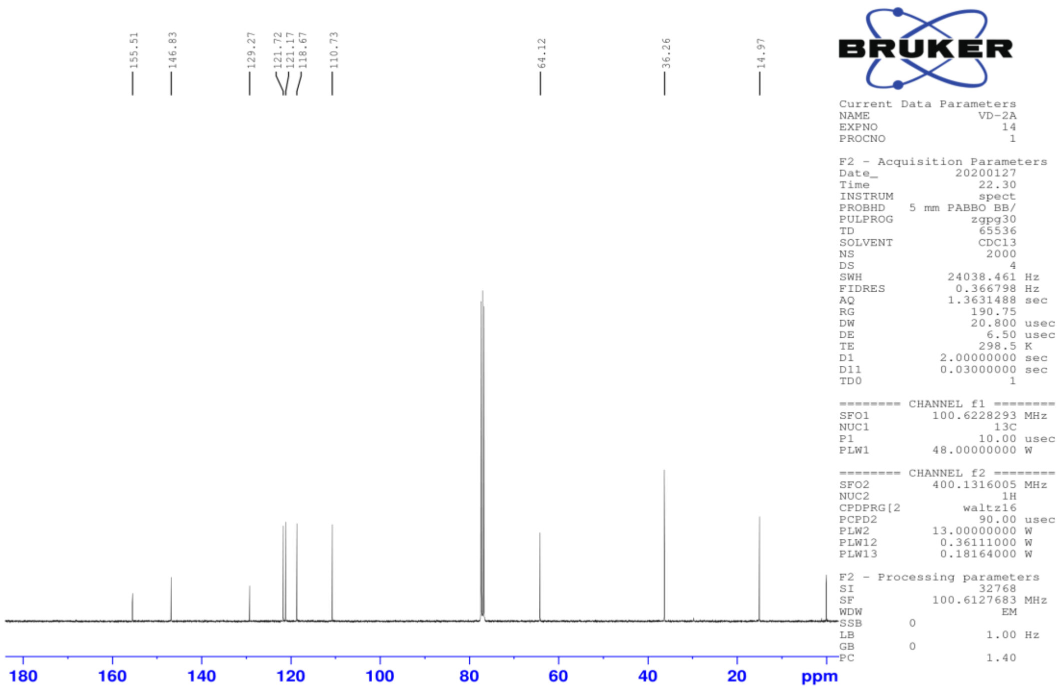

Figure S8. ^{1}H NMR spectrum (**a**) and ^{13}C NMR (**b**) of compound **8** (**2A**).

(a)

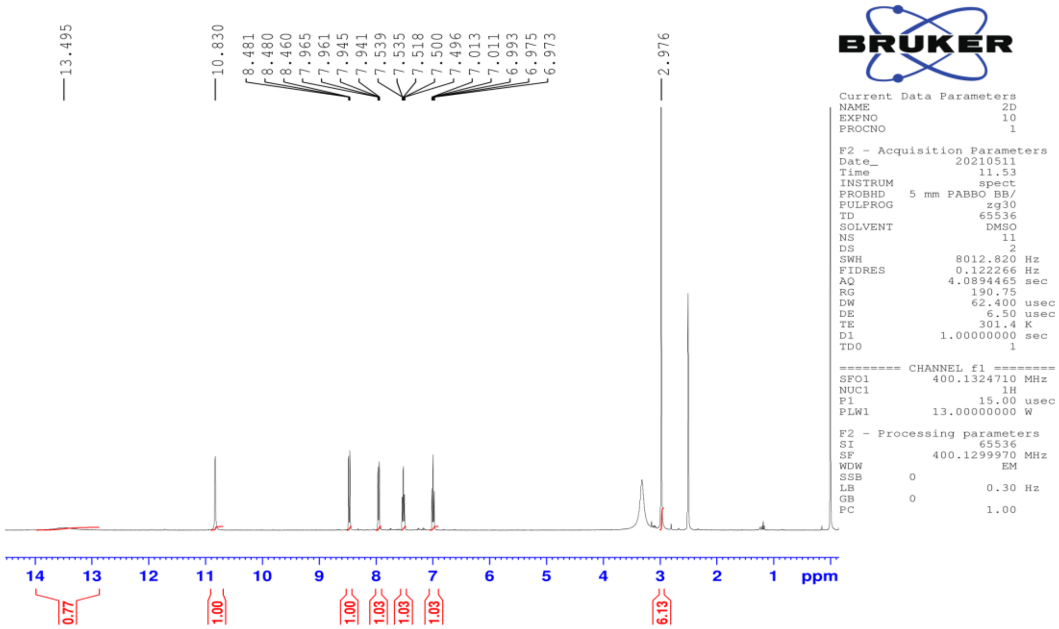

(b)

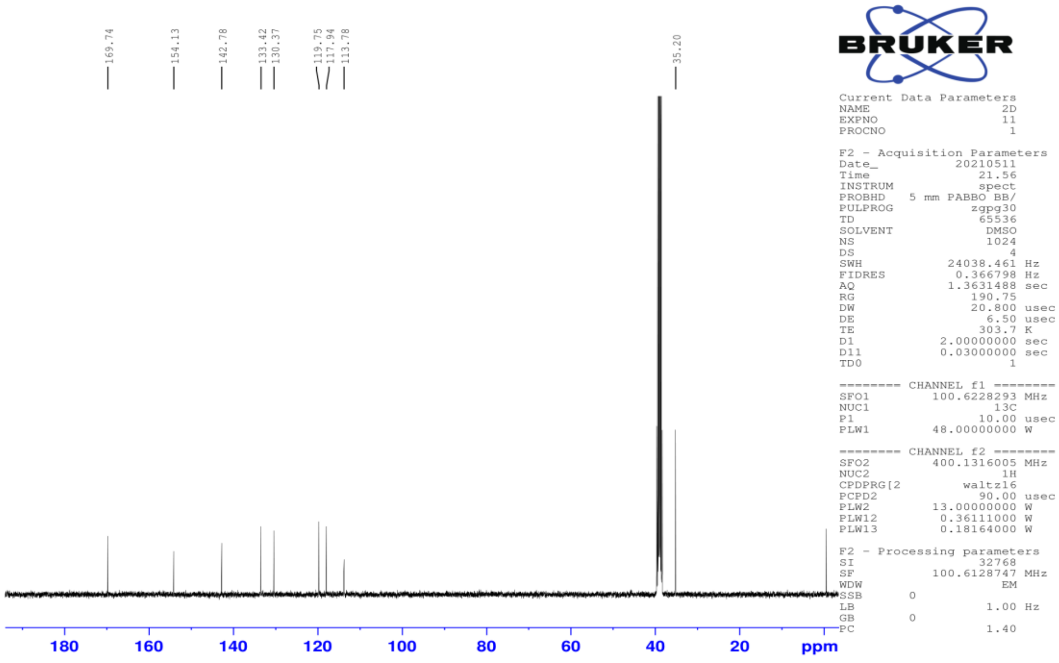

Figure S9. ^{1}H NMR spectrum (**a**) and ^{13}C NMR (**b**) of compound **9** (**2D**).

(a)

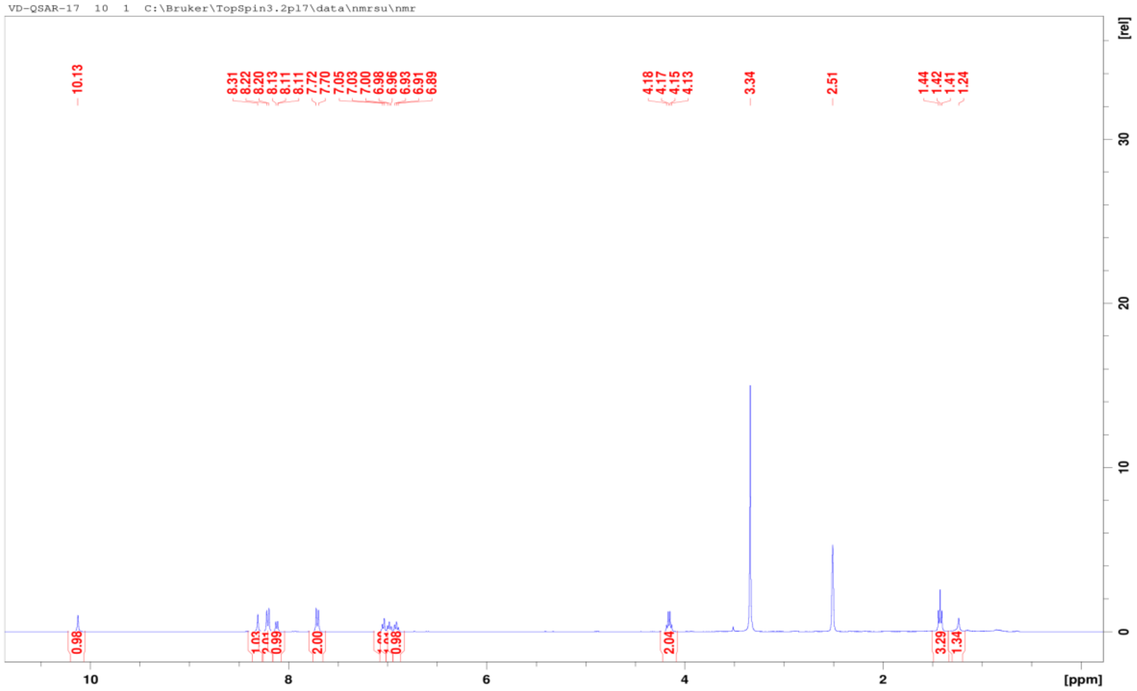

(b)

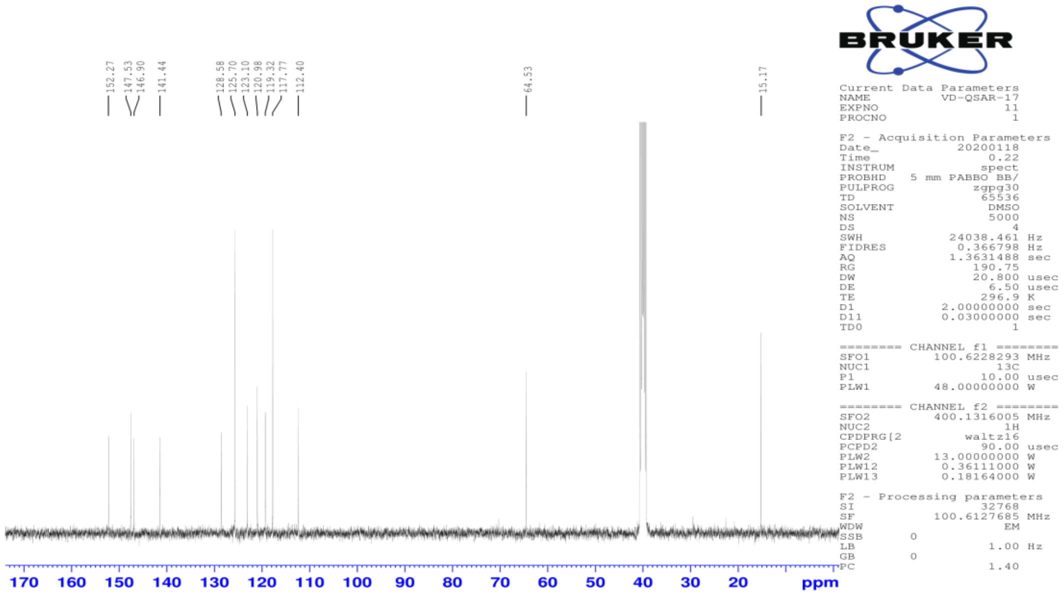

Figure S10. ^{1}H NMR spectrum (**a**) and ^{13}C NMR (**b**) of compound **10** (**QSAR17**).

(a)

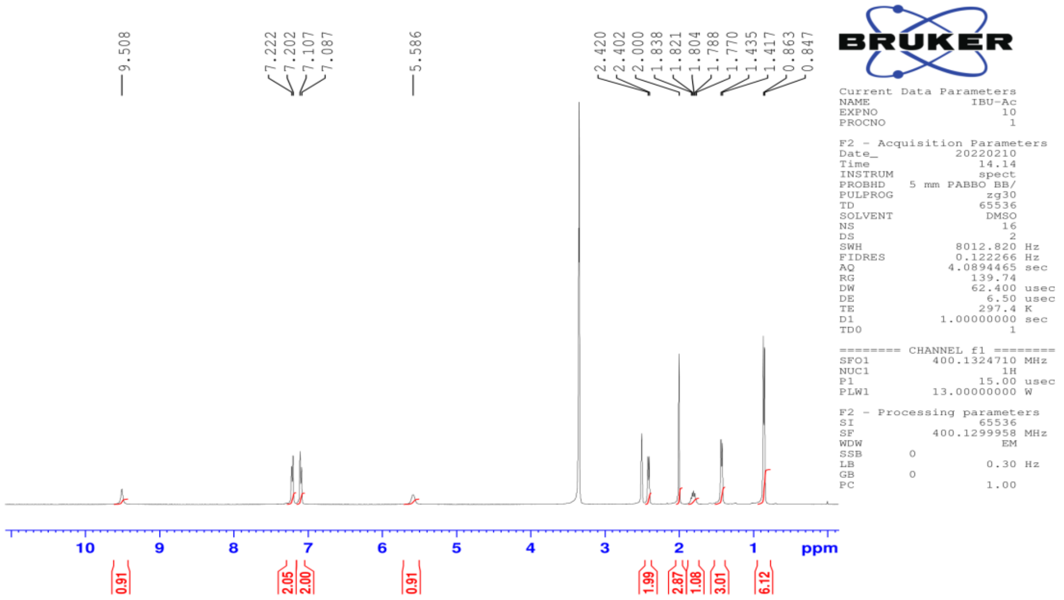

(b)

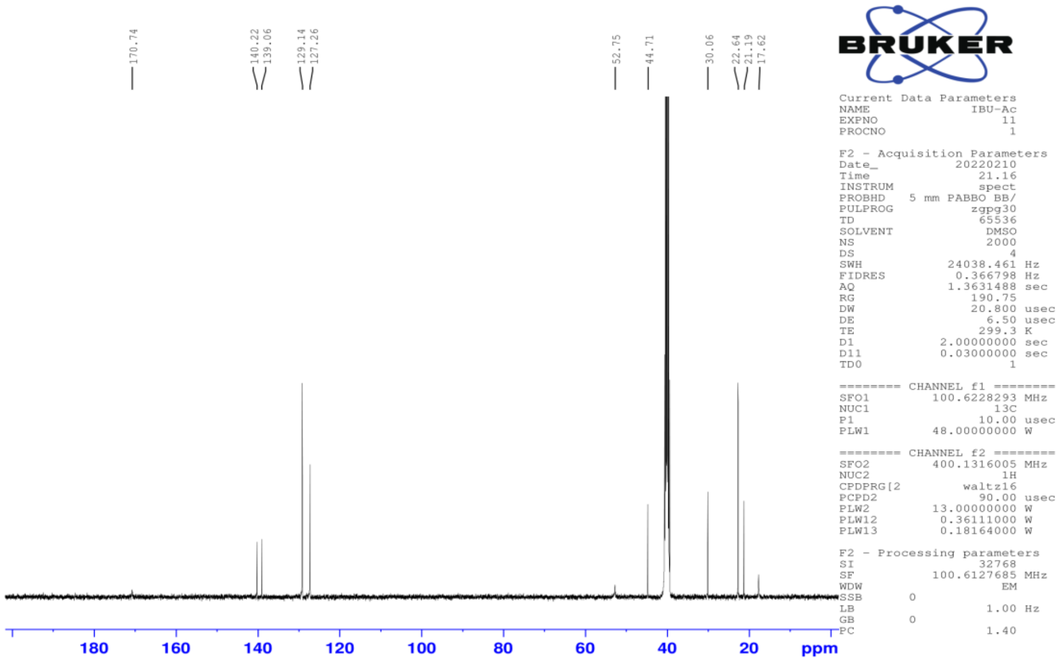

Figure S11. ^{1}H NMR spectrum (**a**) and ^{13}C NMR (**b**) of compound **11** (**IBU-Ac**).

(a)

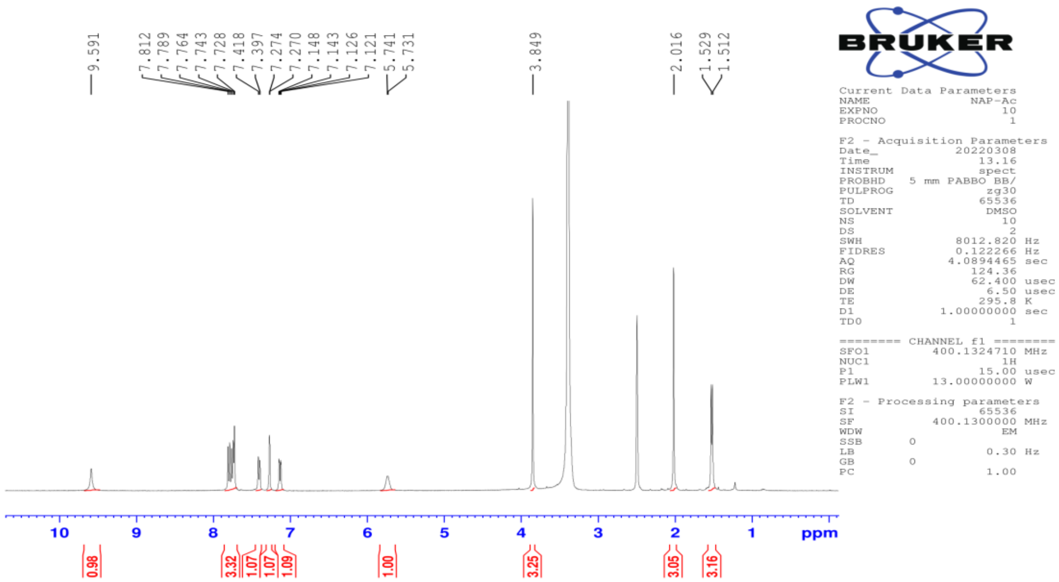

(b)

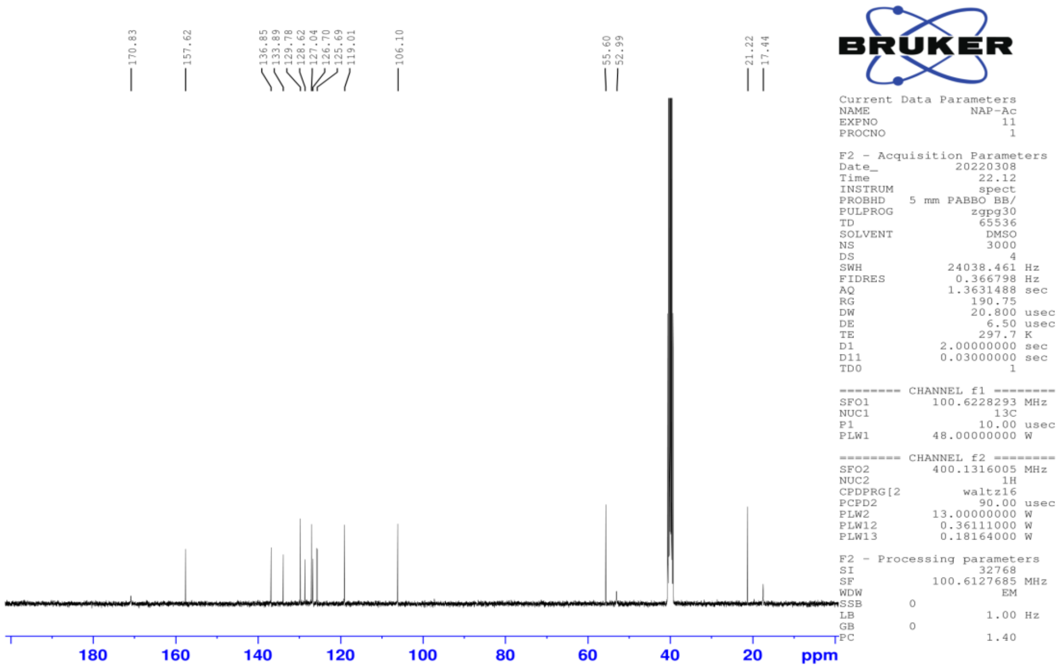

Figure S12. ^{1}H NMR spectrum (**a**) and ^{13}C NMR (**b**) of compound **12** (**NAP-Ac**).

(a)

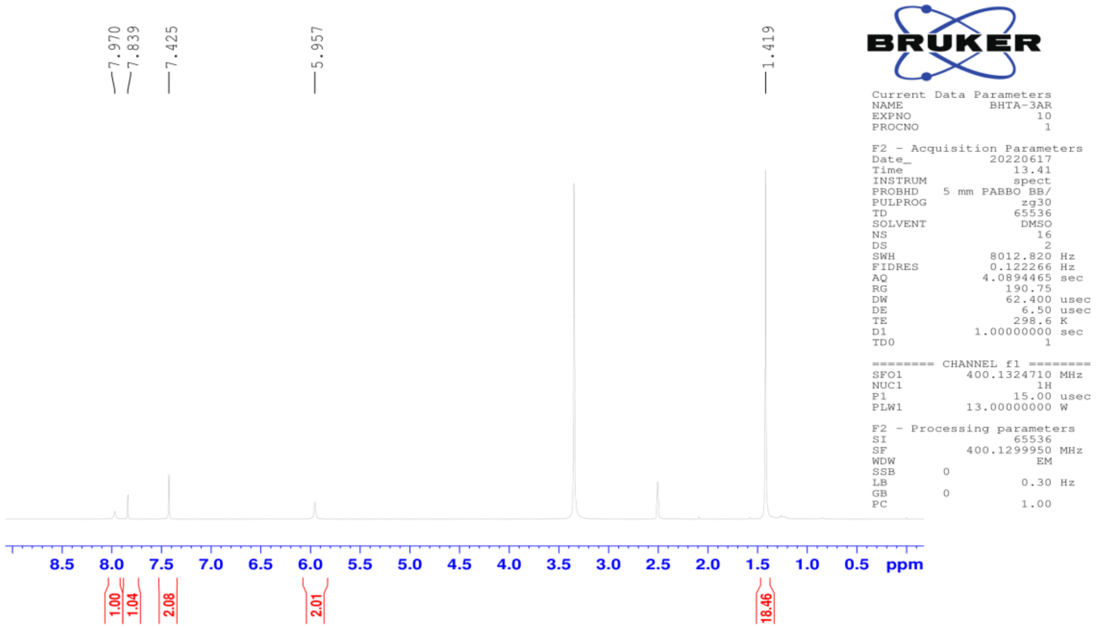

(b)

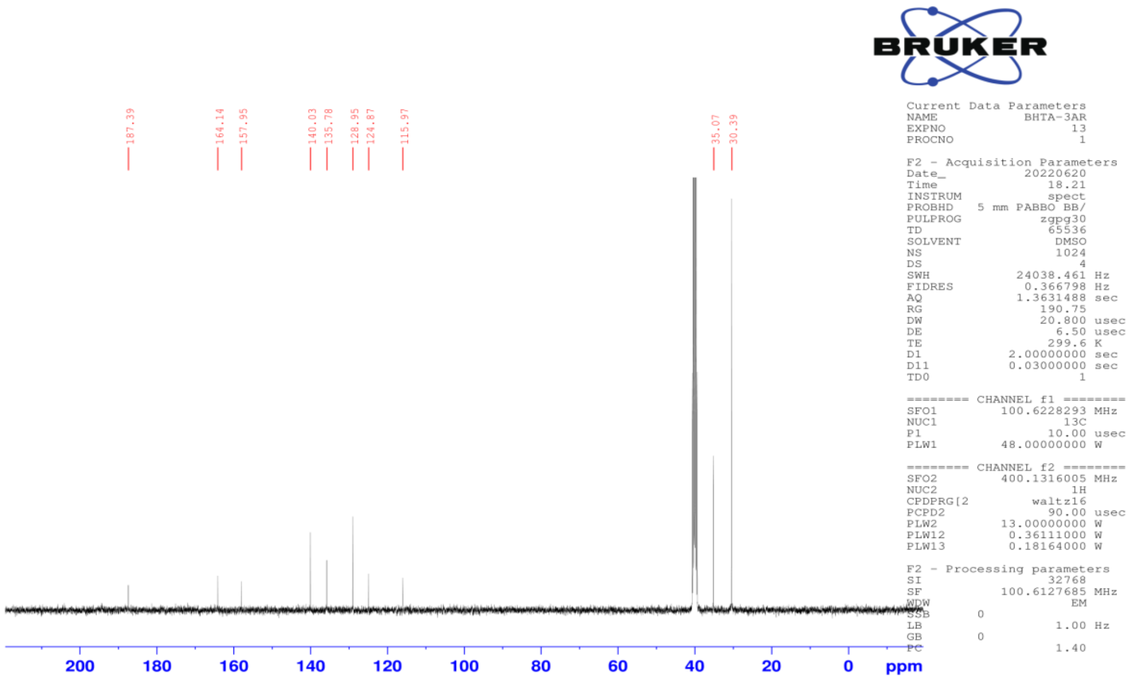

Figure S13. ^{1}H NMR spectrum (**a**) and ^{13}C NMR (**b**) of compound **13** (**BHTA-3AR**).

Tables S1–S4. Statistically significant differences (Wilcoxon test) between tested compounds.

Table S1

								2 h incubation											
	1	2	3	4	5	6	7	8	9	10	11	12	13	BHT	Celecoxib	Zileuton	Urea	Trolox	TBH
1	/	ns	ns	ns	ns	ns	ns	ns	ns	ns	ns	ns	ns	ns	ns	ns	ns	ns	ns
2	ns	/	ns	ns	ns	ns	ns	ns	ns	0.022	0.017	ns	ns	ns	ns	ns	ns	ns	ns
3	ns	ns	/	ns	ns	ns	ns	ns	ns	ns	ns	ns	ns	ns	ns	ns	ns	ns	ns
4	ns	ns	ns	/	ns	ns	ns	ns	ns	0.047	ns	ns	ns	ns	ns	ns	ns	ns	0.005
5	ns	ns	ns	ns	/	ns	ns	ns	ns	ns	ns	ns	ns	ns	ns	ns	ns	ns	0.005
6	ns	ns	ns	ns	ns	/	ns	ns	ns	0.012	ns	ns	ns	ns	ns	ns	ns	ns	ns
7	ns	ns	ns	ns	ns	ns	/	ns	ns	ns	ns	ns	ns	ns	ns	ns	ns	ns	ns
8	ns	ns	ns	ns	ns	ns	ns	/	ns	ns	ns	ns	ns	ns	ns	ns	ns	ns	ns
9	ns	ns	ns	ns	ns	ns	ns	ns	/	ns	ns	ns	ns	ns	ns	ns	ns	ns	0.017
10	ns	0.022	ns	0.047	ns	0.012	ns	ns	ns	/	ns	ns	0.007	ns	ns	ns	ns	ns	0.005
11	ns	0.017	ns	ns	ns	ns	ns	ns	ns	ns	/	ns	ns	ns	ns	ns	ns	ns	0.005
12	ns	ns	ns	ns	ns	ns	ns	ns	ns	ns	ns	/	0.005	ns	ns	ns	ns	ns	ns
13	ns	ns	ns	ns	ns	ns	ns	ns	ns	0.007	ns	0.005	/	ns	ns	ns	ns	ns	0.005
BHT	ns	ns	ns	ns	ns	ns	ns	ns	ns	ns	ns	ns	ns	/	ns	ns	ns	ns	ns
Celecoxib	ns	ns	ns	ns	ns	ns	ns	ns	ns	ns	ns	ns	ns	ns	/	ns	ns	ns	ns
Zileuton	ns	ns	ns	ns	ns	ns	ns	ns	ns	ns	ns	ns	ns	ns	ns	/	ns	ns	ns
Urea	ns	ns	ns	ns	ns	ns	ns	ns	ns	ns	ns	ns	ns	ns	ns	ns	/	ns	ns
Trolox	ns	ns	ns	ns	ns	ns	ns	ns	ns	ns	ns	ns	ns	ns	ns	ns	ns	/	0.006
TBH	ns	ns	ns	0.005	0.005	ns	ns	ns	0.017	0.005	0.005	ns	0.005	ns	ns	ns	ns	0.006	/

Table S2

									24 h incubation										
	1	2	3	4	5	6	7	8	9	10	11	12	13	BHT	Celecoxib	Zileuton	Urea	Trolox	TBH
1	/	ns	ns	0.005	0.012	0.005	ns	ns	ns	ns	0.007	0.005	ns	ns	ns	ns	ns	0.005	0.005
2	ns	/	ns	0.005	0.009	0.005	ns	ns	ns	ns	0.007	0.005	ns	ns	ns	ns	ns	0.047	0.005
3	ns	ns	/	0.005	ns	0.005	ns	ns	ns	ns	ns	0.009	ns	ns	ns	ns	ns	0.028	0.005
4	0.005	0.005	0.005	/	ns	ns	ns	ns	0.022	ns	0.022	ns	0.005	ns	ns	ns	ns	0.005	ns
5	0.012	0.009	ns	ns	/	ns	ns	ns	ns	ns	ns	ns	0.007	ns	ns	ns	ns	0.005	0.007
6	0.005	0.005	0.005	ns	ns	/	ns	ns	ns	ns	0.009	ns	0.005	ns	ns	ns	ns	0.005	ns
7	ns	ns	ns	ns	ns	ns	/	ns	ns	ns	ns	ns	ns	ns	ns	ns	ns	ns	ns
8	ns	ns	ns	ns	ns	ns	ns	/	ns	ns	ns	ns	ns	ns	ns	ns	ns	ns	0.005
9	ns	ns	ns	0.022	ns	ns	ns	ns	/	ns	ns	ns	ns	ns	ns	ns	ns	ns	0.022
10	ns	ns	ns	ns	ns	ns	ns	ns	ns	/	ns	ns	ns	ns	ns	ns	ns	ns	ns
11	0.007	0.007	ns	0.022	ns	0.009	ns	ns	ns	ns	/	0.022	0.007	ns	ns	ns	ns	0.005	0.012
12	0.005	0.005	0.009	ns	ns	ns	ns	ns	ns	ns	0.022	/	0.005	ns	ns	ns	ns	0.005	0.012
13	ns	ns	ns	0.005	0.007	0.005	ns	ns	ns	ns	0.007	0.005	/	ns	ns	ns	ns	ns	0.005
BHT	ns	ns	ns	ns	ns	ns	ns	ns	ns	ns	ns	ns	ns	/	ns	ns	ns	ns	ns
Celecoxib	ns	ns	ns	ns	ns	ns	ns	ns	ns	ns	ns	ns	ns	ns	/	ns	ns	ns	ns
Zileuton	ns	ns	ns	ns	ns	ns	ns	ns	ns	ns	ns	ns	ns	ns	ns	/	ns	ns	ns
Urea	ns	ns	ns	ns	ns	ns	ns	ns	ns	ns	ns	ns	ns	ns	ns	ns	/	ns	ns
Trolox	0.005	0.047	0.028	0.005	0.005	0.005	ns	ns	ns	ns	0.005	0.005	ns	ns	ns	ns	ns	/	0.000
TBH	0.005	0.005	0.005	ns	0.007	ns	ns	0.005	0.022	ns	0.012	0.012	0.005	ns	ns	ns	ns	0.000	/

Table S3

								2 h incubation, TBH added											
	1	2	3	4	5	6	7	8	9	10	11	12	13	BHT	Celecoxib	Zileuton	Urea	Trolox	TBH
1	/	0.012	ns	ns	ns	ns	ns	ns	ns	ns	ns	0.022	ns	ns	ns	ns	ns	0.009	ns
2	0.012	/	ns	ns	ns	ns	ns	0.007	0.028	0.012	ns	ns	0.022	ns	ns	ns	ns	ns	0.050
3	ns	ns	ns	ns	ns	ns	ns	0.028	ns	ns	ns	ns	ns	ns	ns	ns	ns	0.028	ns
4	ns	ns	ns	/	0.007	ns	ns	ns	ns	ns	ns	ns	ns	ns	ns	ns	ns	0.005	ns
5	ns	ns	ns	0.007	/	ns	ns	0.047	ns	0.037	ns	ns	ns	ns	ns	ns	ns	ns	ns
6	ns	ns	ns	ns	ns	/	ns	ns	ns	0.047	ns	ns	ns	ns	ns	ns	ns	ns	ns
7	ns	ns	ns	ns	ns	ns	/	ns	ns	ns	ns	ns	ns	ns	ns	ns	ns	ns	ns
8	ns	0.007	0.028	ns	0.047	ns	ns	/	ns	ns	0.017	0.007	0.028	ns	ns	ns	ns	0.022	ns
9	ns	0.028	ns	ns	ns	ns	ns	ns	/	ns	ns	0.047	ns	ns	ns	ns	ns	0.037	ns
10	ns	0.012	ns	ns	0.037	0.047	ns	ns	ns	/	ns	0.007	ns	ns	ns	ns	ns	0.028	ns
11	ns	ns	ns	ns	ns	ns	ns	0.017	ns	ns	/	ns	ns	ns	ns	ns	ns	0.037	ns
12	0.022	ns	ns	ns	ns	ns	ns	0.007	0.047	0.007	ns	/	0.009	ns	ns	ns	ns	ns	0.028
13	ns	0.022	ns	ns	ns	ns	ns	0.028	ns	ns	ns	0.009	/	ns	ns	ns	ns	ns	ns
BHT	ns	ns	ns	ns	ns	ns	ns	ns	ns	ns	ns	ns	ns	/	ns	ns	ns	ns	ns
Celecoxib	ns	ns	ns	ns	ns	ns	ns	ns	ns	ns	ns	ns	ns	ns	/	ns	ns	ns	ns
Zileuton	ns	ns	ns	ns	ns	ns	ns	ns	ns	ns	ns	ns	ns	ns	ns	/	ns	ns	ns
Urea	ns	ns	ns	ns	ns	ns	ns	ns	ns	ns	ns	ns	ns	ns	ns	ns	/	ns	ns
Trolox	0.009	ns	0.028	0.005	ns	ns	ns	0.022	0.037	0.028	0.037	ns	ns	ns	ns	ns	ns	/	0.006
TBH	ns	0.050	ns	ns	ns	ns	ns	ns	ns	ns	ns	0.028	ns	ns	ns	ns	ns	0.006	/

Table S4

								24 h incubation, TBH added											
	1	2	3	4	5	6	7	8	9	10	11	12	13	BHT	Celecoxib	Zileuton	Urea	Trolox	TBH
1	/	ns	ns	ns	ns	ns	ns	0.005	ns	ns	0.005	ns	ns	ns	ns	ns	ns	0.005	ns
2	ns	/	0.022	ns	ns	ns	ns	0.007	ns	ns	0.012	ns	ns	ns	ns	ns	ns	0.005	ns
3	ns	0.022	/	0.005	0.017	0.022	ns	ns	ns	ns	ns	0.037	ns	ns	ns	ns	ns	0.005	0.037
4	ns	ns	0.005	/	0.047	ns	ns	0.007	ns	ns	0.005	ns	0.028	ns	ns	ns	ns	0.005	ns
5	ns	ns	0.017	0.047	/	ns	ns	0.005	ns	ns	0.005	ns	ns	ns	ns	ns	ns	0.005	ns
6	ns	ns	0.022	ns	ns	/	ns	0.005	ns	ns	0.005	ns	0.028	ns	ns	ns	ns	0.005	ns
7	ns	ns	ns	ns	ns	ns	/	ns	ns	ns	ns	ns	ns	ns	ns	ns	ns	ns	ns
8	0.005	0.007	ns	0.007	0.005	0.005	ns	/	ns	0.028	0.009	0.007	0.005	ns	ns	ns	ns	ns	0.012
9	ns	ns	ns	ns	ns	ns	ns	ns	/	ns	ns	ns	ns	ns	ns	ns	ns	ns	0.022
10	ns	ns	ns	ns	ns	ns	ns	0.028	ns	/	ns	ns	ns	ns	ns	ns	ns	ns	ns
11	0.005	0.012	ns	0.005	0.005	0.005	ns	0.009	ns	ns	/	0.007	ns	ns	ns	ns	ns	0.005	0.028
12	ns	ns	0.037	ns	ns	ns	ns	0.007	ns	ns	0.007	/	ns	ns	ns	ns	ns	0.005	ns
13	ns	ns	ns	0.028	ns	0.028	ns	0.005	ns	ns	ns	ns	/	ns	ns	ns	ns	0.005	0.047
BHT	ns	ns	ns	ns	ns	ns	ns	ns	ns	ns	ns	ns	ns	/	ns	ns	ns	ns	ns
Celecoxib	ns	ns	ns	ns	ns	ns	ns	ns	ns	ns	ns	ns	ns	ns	/	ns	ns	ns	ns
Zileuton	ns	ns	ns	ns	ns	ns	ns	ns	ns	ns	ns	ns	ns	ns	ns	/	ns	ns	ns
Urea	ns	ns	ns	ns	ns	ns	ns	ns	ns	ns	ns	ns	ns	ns	ns	ns	/	ns	ns
Trolox	0.005	0.005	0.005	0.005	0.005	0.005	ns	ns	ns	ns	0.005	0.005	0.005	ns	ns	ns	ns	/	0.000
TBH	ns	ns	0.037	ns	ns	ns	ns	0.012	0.022	ns	0.028	ns	0.047	ns	ns	ns	ns	0.000	/
